# Chaihu-shugan-san alleviates depression-like behavior in mice exposed to chronic unpredictable stress by altering the gut microbiota and levels of the bile acids hyocholic acid and 7-ketoDCA

**DOI:** 10.3389/fphar.2022.1040591

**Published:** 2022-10-19

**Authors:** Chong Ma, Dun Yuan, Stephen James Renaud, Ting Zhou, Fan Yang, Yuligh Liou, Xinjian Qiu, Lu Zhou, Ying Guo

**Affiliations:** ^1^ Department of Clinical Pharmacology, Xiangya Hospital, Central South University, Changsha, China; ^2^ Hunan Key Laboratory of Pharmacogenetics, Institute of Clinical Pharmacology, Central South University, Changsha, China; ^3^ Engineering Research Center of Applied Technology of Pharmacogenomics, Ministry of Education, Changsha, China; ^4^ National Clinical Research Center for Geriatric Disorders, Xiangya Hospital, Central South University, Changsha, China; ^5^ School of Pharmaceutical Sciences (Shenzhen), Sun Yat-sen University, Guangzhou, China; ^6^ Department of Neurosurgery, Xiangya Hospital, Central South University, Changsha, China; ^7^ Department of Anatomy and Cell Biology, The University of Western Ontario, London, ON, Canada; ^8^ China Xiangya Medical Laboratory, Central South University, Changsha, China; ^9^ Department of Integrated Traditional Chinese and Western Medicine, Xiangya Hospital, Central South University, Changsha, China

**Keywords:** Chaihu-Shugan-San, depression, bile acids, *Parabacteroides distasonis*, gut microbiota-brain axis

## Abstract

Chaihu-Shugan-San (CSS) is a traditional botanical drug formula often prescribed to treat depression in oriental countries, but its pharmacotherapeutic mechanism remains unknown. It was recently reported that CSS alters the composition of intestinal microflora and related metabolites such as bile acids (BAs). Since the intestinal microflora affects physiological functions of the brain through the gut-microbiota-brain axis, herein we investigated whether CSS altered BA levels, gut microflora, and depression-like symptoms in chronic unpredictable mild stress (CUMS) mice, a well-established mouse model of depression. Furthermore, we determined whether BA manipulation and fecal microbiota transplantation altered CSS antidepressant actions. We found that the BA chelator cholestyramine impaired the antidepressant effects of CSS, which was partially rescued by dietary cholic acid. CSS increased the relative abundance of *Parabacteroides distasonis* in the colon of CUMS mice, and increased serum levels of various BAs including hyocholic acid (HCA) and 7-ketodeoxycholic acid (7-ketoDCA). Furthermore, gut bacteria transplantation from CSS-treated mice into untreated or cholestyramine-treated CUMS mice restored serum levels of HCA and 7-ketoDCA, alleviating depression-like symptoms. In the hippocampus, CSS-treated mice had decreased expression of genes associated with BA transport (Bsep and Fxr) and increased expression of brain-derived neurotrophic factor and its receptor, TrkB. Overall, CSS increases intestinal *P. distasonis* abundance, leading to elevated levels of secondary BAs in the circulation and altered expression of hippocampal genes implicated in BA transport and neurotrophic signaling. Our data strongly suggest that the gut microbiota-brain axis contributes to the potent antidepressant action of CSS by modulating BA metabolism.

## Introduction

Depression is a serious neuropsychiatric disorder characterized by persistent depressed mood and lack of pleasure, and is the world’s leading cause of years lost due to disability (Huang et al., 2019; O'Leary, 2021). Approximately 260 million people worldwide suffer from depression, and this number is on the rise (Zhou et al., 2020). The pathogenesis of depression is complex and involves physiological, psychological, and environmental factors (Spichak et al., 2021). Imbalances in the levels of monoamine neurotransmitters or neurotrophic factors are the main hypotheses for the pathogenesis of depression, based primarily on deficiencies in biogenic amine systems or brain-derived neurotrophic factor (BDNF) signaling, respectively (Ma et al., 2021a; Li et al., 2021). However, only 50% of patients respond to an initial antidepressant treatment, and less than 40% of patients achieve remission (Rush et al., 2006; Bennabi et al., 2015). Therefore, the discovery of new targets to treat depression more effectively is of great importance.

The gut-brain axis consists of bidirectional communication between the central nervous system and the gastrointestinal tract. Gut microbes produce numerous metabolites such as short-chain fatty acids and bile acids (BAs) that enter the circulation and facilitate the exchange of information between the gut and brain (Spichak et al., 2021). Accumulating evidence shows a discrepancy in the composition of gut microflora between healthy individuals and patients suffering from depression, underscoring the importance of the gut microbiome for normal brain physiology (Zheng et al., 2016; Han and Kim, 2019; Yu et al., 2019; Xie et al., 2020). Therefore, investigating how metabolites produced by intestinal flora, such as BAs, are involved in normal brain functioning and the pathogenesis of depression may uncover new opportunities for therapeutic intervention.

BAs are a class of amphoteric steroids produced in the liver and metabolized by intestinal microorganisms, which can then reenter circulation and have pleiotropic roles as endogenous signaling mediators (Stanimirov et al., 2012; Guo et al., 2016). Cholic acid (CA), which comprises approximately 22% of all BAs in humans and 37% in mice, is a primary BA manufactured by the liver from cholesterol, which is then metabolized to secondary BAs by intestinal flora (Guo et al., 2016; Ma et al., 2021b). For instance, CA can be converted into 7-ketodeoxycholic acid (7-ketoDCA) by the action of bacteria such as *Parabacteroides distasonis*. Chenodeoxycholic acid (CDCA), another primary BA produced by the liver, can be converted by intestinal microflora to hyocholic acid (HCA) by 6-hydroxylation (Spinelli et al., 2016; Wang et al., 2019). Thus, gut microbiota are critical regulators of BA metabolism and diversity.

Altered BA levels have been implicated in the development of various neurological disorders including Alzheimer’s disease, Parkinson’s syndrome, and depression (MahmoudianDehkordi et al., 2019; Shao et al., 2021). For instance, studies have shown that total serum BA levels are decreased in patients with major depressive disorder compared to healthy volunteers (Peng et al., 2016; Lu et al., 2018; Cheng et al., 2019); however, the involvement of BAs in the etiology and pathogenesis of depression is still unclear. BAs are endogenous ligands for farnesoid X receptor (FXR), a nuclear receptor that plays an important role in cholesterol and BA homeostasis and regulates expression of genes involved in cellular metabolism (Parks et al., 1999). Recent studies using mice have found that activation of FXR in the hippocampus inhibits expression of genes encoding BDNF and its receptor, tropomyosin-related kinase B (TrkB) (Seok et al., 2014; Hu et al., 2020). However, whether BAs influence depressive behavior in mice has not been reported.

Chaihu-Shugan-San (CSS) is a solution consisting of 7 crude botanical drugs, namely Chai Hu (*Bupleurum chinense DC*.), Chen Pi (*Citrus reticulata Blanco*), Xiang Fu (*Cyperus rotundus L.*), Zhi Qiao (*Citrus × aurantium L.*), Chuan Xiong (*Ligusticum striatum DC.*), Bai Shao (*Paeonia lactiflora Pall.*), and Gan Cao (*Glycyrrhiza uralensis Fisch.*) in a specific ratio of 4: 4: 3: 3: 3: 3: 1 (Supplementary Table S1). All plant names and species were validated using http://www.theplantlist.org/ (Xie et al., 2013; Heinrich et al., 2020). It is commonly used in the treatment of clinical depression in China, and several meta-analyses have reported that CSS has fewer side effects and higher efficacy for treating depression than commonly-used antidepressants such as selective serotonin reuptake inhibitors (Wang et al., 2012; Butler and Pilkington, 2013; Yeung et al., 2014; Zhu et al., 2021). CSS affects multiple pathways that may contribute to its antidepressant actions, including regulating the expression of 5-HT1A receptors and reducing neuroinflammation (Kim et al., 2005; Chen et al., 2018a; Jia et al., 2018). Studies using non-targeted metabolomics have identified increased relative levels of CA following CSS treatment in animal models of chronic stress (Su et al., 2014; Jia et al., 2017), although absolute quantification of changes in BA profile after CSS intervention has not yet been determined. Furthermore, the contribution of BAs to the antidepressant functions of CSS is unknown. We therefore hypothesized that CSS-mediated changes in gut microflora composition and BA levels are key mechanisms contributing to its antidepressant effect. Herein, we confirm that CSS alleviates depression-like symptoms in mice exposed to chronic unpredictable mild stress (CUMS), and that serum BA profiles, gut microflora composition, and expression of genes encoding neurotrophic factors in the hippocampus are restored in CSS-treated mice to levels similar to controls. These CSS-induced effects were inhibited in mice treated with cholestyramine (CHOL), which sequesters BAs in the intestine, and were recapitulated by transplanting fecal microbiota from CSS-treated mice into untreated mice. Our findings strongly suggest that CSS exerts antidepressant effects by modulating the constitution of intestinal microbiota to promote BA synthesis and metabolism.

## Materials and methods

### Chemicals and reagents

Reference standards of 42 BAs and 4 deuterated internal standards were acquired from ZZBIO Co., Ltd. (Shanghai, China) including 3-oxocholic acid (3-oxoCA), 6-ketolithocholic acid (6-ketoLCA), 7-ketoDCA, 7-ketoLCA, allocholic acid (ACA), allocholic acid (apoCA), CA, CDCA, deoxycholic acid (DCA), HCA, dehydrocholic acid (DHCA), dehydrolithocholic acid (DHLCA), hyodeoxycholic acid (HDCA), isoallolithocholic acid (isoalloLCA), isolithocholic acid (isoLCA), lithocholic acid (LCA), 3-sulfoglylithocholic acid (LCA-3S), ursocholic acid (UCA), ursodeoxycholic acid (UDCA), α muricholic acid (αMCA), βMCA, ωMCA, Glyco-CA (GCA), GCDCA, GDCA, GDHCA, GHCA, GHDCA, GLCA, GLCA-3S, GUDCA, Tauro-CA TCA, TCDCA, TDCA, TDHCA, THCA, THDCA, TLCA, TLCA-3S, TUDCA, TαMCA, TβMCA, GCA-2,2,4,4-d4 (D_4_-GCA), TCA-2,2,4,4-d4 (D_4_-TCA), CA-2,2,4,4-d4 (D_4_-CA), and LCA-2,2,4,4-d4 (D_4_-LCA). HPLC-grade methanol, acetonitrile, water, ammonium acetate, and activated carbon were obtained from Sigma–Aldrich (St. Louis, MO, United States).

### Preparation of Chaihu-shugan-san extract

The composition and corresponding ratio of CSS are listed in Supplementary Table S1. All botanical drugs required to prepare CSS were purchased from Hunan Zhenxing Chinese Medicine Co., Ltd. (Hunan, China. Drug Manufacturing Certificate: NO.20150,021, Drug GMP certificate: HN20150,147). The authentication of the purchased herbs was performed by Professor Peng Lei, Department of Chinese herbal medicine of Central South University (CSU, Changsha, China).

Preparation of CSS extract and compositional analysis was performed as described (Qiu et al., 2011; Zhu et al., 2021). Briefly, the raw herbal ingredients of CSS were mixed as described previously and submerged into powder. The mixture was soaked in distilled water for 30 min, and then condensate reflux was extracted three times for 30 min. The filtrates were collected and condensed at 70°C in a vacuum. The final yield of CSS was 29.54%. Details of the UPLC procedure that was used to measure the composition of CSS extract are provided in Supplemental Material.

### Animals and treatments

Seven-week-old male C57BL/6J mice were purchased from Hunan SJA Laboratory Animal Co. Ltd. (Hunan, China). All mice were maintained under specific pathogen-free conditions and housed with free access to sterile food and water, under a standard 12-h dark-light cycle and humidity-controlled environment with room temperature at approximately 25°C. Mice were allowed, and to acclimated for at least 1 week before treatment. According to the results of the sucrose preference test, the mice were randomly divided into groups by SPSS software. During experiments mice were housed alone to ensure consistency across groups.

In the first series of experiments, mice (*n* = 8-9 per group) were divided into 5 groups: 1) a control group: not exposed to CUMS but received vehicle (saline) intragastrically (i.g.) once daily for 8 weeks; 2) NC group: exposed to CUMS and received saline i. g.; 3) CSS group: exposed to CUMS and received 20 mg/kg/d CSS i. g.; 4) CSS + CHOL group: exposed to CUMS and received 20 mg/kg/d CSS i. g. as well as 2% CHOL in the feed; and 5) CSS + CHOL + CA group: exposed to CUMS and received 20 mg/kg/d CSS i. g., as well as 2% CHOL and 1% CA in the feed, 2% CHOL and 1% CA rodent diet were provided at intervals days. Mice in all groups were sacrificed after 8 weeks, and serum, liver, colon contents, and hippocampus were collected for further analysis.

In the next series of experiments involving fecal microbiota transplantation (FMT) from CSS-treated mice into untreated mice, mice were divided into 7 groups (*n* = 7-8 mice per group): 1) Control group (as above); 2) NC group (as above); 3) CSS group (as above); 4) CSS + CHOL group (as above); 5) CSS + CHOL + FMT group: exposed to CUMS and received 20 mg/kg/d CSS i. g. As well as 2% CHOL in the feed. Meanwhile, fecal suspension prepared from the CSS group (group 3) was then transplanted into these mice; 6) FFMT group: mice exposed to CUMS received mixed antibiotics (containing 0.5 g/L vancomycin, 1 g/L neomycin sulfate, 1 g/L metronidazole and 1 g/L ampicillin) in drinking water for 1 week. Afterwards, fecal suspension from the CSS group was filtered through a 0.22 μm filter (to remove microbials) and transplanted into these mice; 7) FMT group: same as group 6 above except the fecal suspension was not filtered prior to transplantation. The procedure for our patented FMT/FFMT method is shown in Supplementary Figure S1. For all groups, mice were sacrificed after 8 weeks, and the serum and colon contents were collected for further analysis.

All experimental procedures and animal care were carried out in compliance with the regulations of the Animal Care Committee of Central South University (CTXY-150001–15).

### Chronic unpredictable mild stress procedure

The CUMS protocol was conducted as previously described (Yan et al., 2020a). Briefly, mice were randomly exposed to two of the following stresses once daily for 8 weeks: cage tilting (45°) for 12 h, wet the pad for 15 h, water deprivation for 24 h, reverse light-dark cycle for 24 h, forced swimming in cold water for 3 min, tail suspension for 3 min, nipped tail for 1 min, strange object for 4 h, or physical restraint for 5 h. Except for the control group, animals were randomly exposed to only two kinds of stimulation every day. Mice were not exposed to the same stress for more than two consecutive days.

### Behavioral testing

***Sucrose Preference Test.*** Mice were trained for 72 h before the test. Briefly, two bottles containing 1% sucrose were given to mice for 24 h, and then the solution was replaced in one of the bottles with pure water for another 24 h. Mice were then deprived of water and food for 12 h. After this fasting period, one bottle containing 1% sucrose and one bottle containing pure water (100 ml each) were provided, and the consumption of 1% sucrose and pure water was measured for 1 h to calculate the sucrose preference index according to the formula: sucrose preference percent = consumption of 1% sucrose/(consumption of 1% sucrose + consumption of pure water) × 100.

***Forced Swimming Test.*** Mice were placed in a glass beaker (10 cm high) filled to the 8-cm mark with water (25 ± 1°C). Mice were allowed to acclimatize for 6 min. Swimming behavior, including struggle (positive time) and immobility (negative time) was then recorded for the next 4 min. The water was changed following each test.

### UHPLC-QQQ MS analysis for bile acid quantification

To measure BA levels in serum and liver, a UHPLC-QQQ MS analysis for 42 endogenous BAs was performed. Details regarding the preparation of samples and validation procedures are provided in Supplementary Tables S2–S9. Method validation procedures closely adhered to the FDA’s Guidance for Bioanalytical Method Validation for Industry to test the limit of detection, lower limit of quantification, linearity, intra- and inter-assay precision, accuracy, matrix effects, and extraction recovery.

### Reverse transcriptase quantitative polymerase chain reaction

Total RNA was isolated from cells using TRIzol reagent (Takara, Shiga, Japan) according to the manufacturer’s instructions, and 1 μg of total RNA was reverse transcribed using the PrimeScript RT Reagent Kit (Takara). Reverse transcriptase quantitative PCR (RT-qPCR) was performed using SYBR Premix Ex Tap (Takara) and run on an LC480 instrument (Roche Diagnostics, Meylan, France). The 2^−ΔΔCt^ method was used to quantify gene expression of *Oatp1a1*, *Oatp1a4*, *Bsep, Fxr*, *Bdnf*, and *Trkb*, with expression of *Gapdh* used for normalization. Colon content samples and DNA were extracted using the QIAamp Fast DNA Stool Mini kit (Qiagen Diagnostics, Germany). The relative abundance of *P. distasonis* was normalized to that of universal bacteria. The primer sequences are listed in Table S10.

### 16S rRNA sequencing

Fresh colon contents were collected, placed into a 15 ml tube with PBS, and stored at −80°C. Bacterial DNA was extracted using an E. Z. N. A. Stool DNA kit (Omega bio-tek, Norcross, GA, United States). The 16S rRNA sequence embedded within bacterial DNA was sequenced using a ThermoFisher Ion S5TMXL (service provided by Novogene Corporation, Beijing, China) following the manufacturer’s guidelines. The merged raw tags were filtered and developed into clean tags according to the Cutadapt (Version 1.9.1) quality control process, and the chimera sequence was detected using the UCHIME algorithm (Version 11). The clean tags were clustered into operational taxonomic units (OTUs) with 97% similarity by Uparse (Version 7.0.1001). The representative sequence for each OTU was selected, and the taxonomic information was annotated using the SILVA database (Release 132) by the Mothur method. The OTU and taxonomic data were used for further analysis.

### Statistical analysis

Data are expressed as means ± standard deviation (S.D.; *n* = 7-9 mice per group). Statistical analyses were performed with an IBM-SPSS 21.0 computer program (IBM, Armonk, NY), STAMP 2.1.3 software, and R studio version 4.0.2. One-way analysis of variance (ANOVA) followed by Duncan’s post-hoc test was used to analyze differences between groups. Principal component analyses (PCA) were generated by STAMP 2.1.3 software. Statistical significance was set at *p* < 0.05 for all analyses.

## Results

### Chaihu-shugan-san alleviates depression-like symptoms in chronic unpredictable mild stress mice through a bile acid-dependent mechanism

**FIGURE 1:**
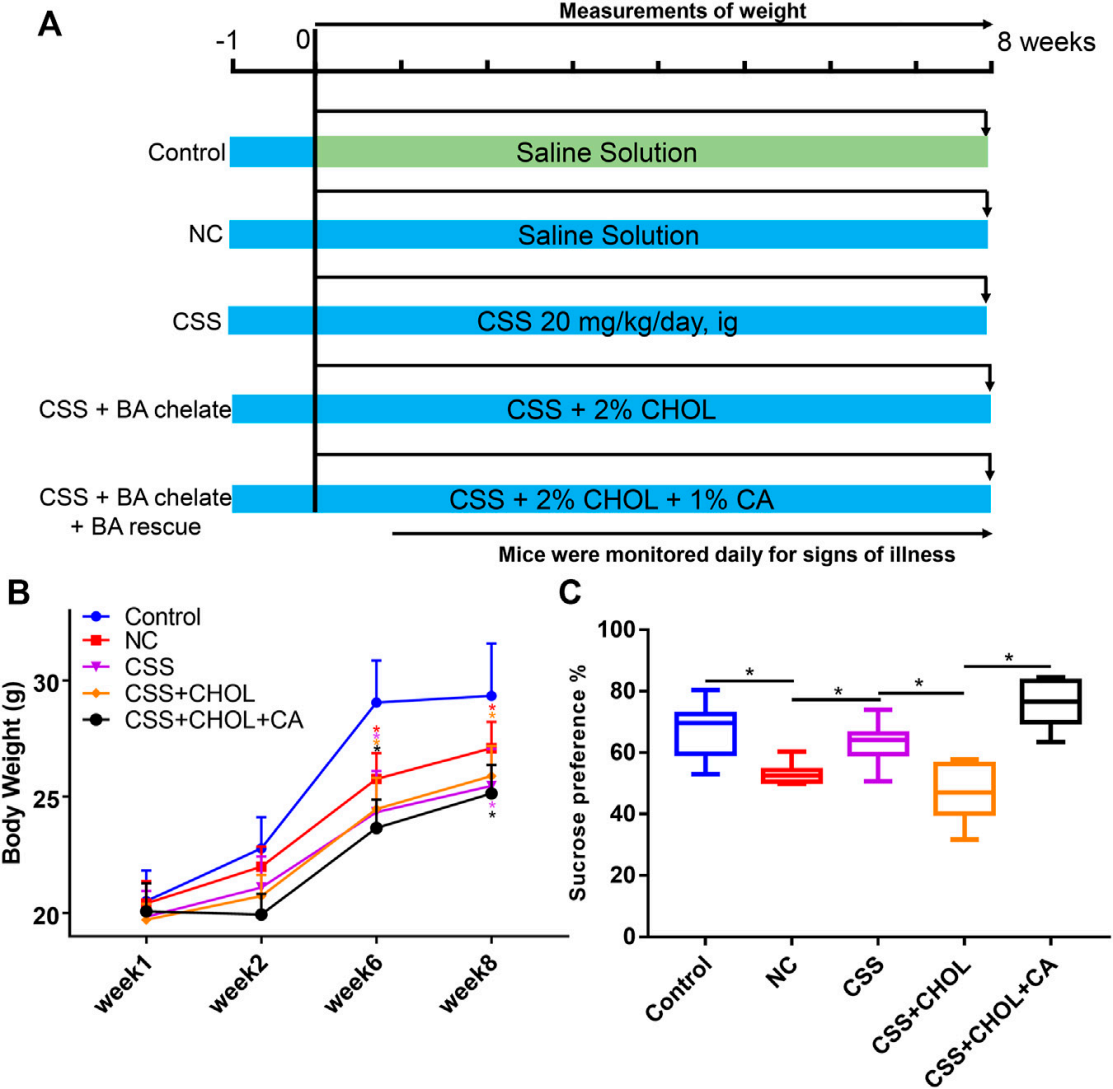
Effect of CSS and BA manipulation on body weight and sucrose preference in a CUMS mouse model of depression. Mice were exposed to CUMS and treated with saline (NC group), CSS, or CSS along with the BA sequestrant CHOL with or without CA supplementation. Control mice were not exposed to CUMS but received saline. **(A)** Overview of experimental groups and timelines. **(B)** Body weights of mice in each group measured 1, 2, 6, and 8 weeks after starting the experiment. **(C)** Percent sucrose preference (based on consumption of 1% sucrose in the sucrose preference test). All values are expressed as the mean ± S.D. An asterisk “*” indicates *p* < 0.05 (*n* = 8-9 mice per group).

To explore the role of BAs in the antidepressant efficacy of CSS *in vivo*, we administered CHOL to inhibit intestinal BA reabsorption in a CUMS-induced mouse model of depression. CHOL is a non-absorbable quaternary amine-based anion exchange resin that binds to and sequesters BAs in the intestine, thereby decreasing reabsorption of BAs. Therefore, we hypothesized that reduced BA reabsorption due to administration of CHOL would reduce the antidepressant efficacy of CSS in CUMS mice. Compared with the control group, body weights of mice in all groups exposed to the CUMS protocol, including NC, CSS, CSS + CHOL, and CSS + CHOL + CA, were significantly decreased at 6 and 8 weeks (Figures 1A,B, *p* < 0.05). Mice in the NC group had a lower sucrose preference index in addition to decreased positive time and increased immobility time in the forced swim test compared to controls, suggesting that the CUMS protocol elicited depression-like symptoms in these mice (*p* < 0.05; Figure 1C, Supplementary Figure S2). Mice exposed to the CUMS protocol while receiving CSS had improved sucrose preference compared with the NC group, which was expected given the antidepressant properties of CSS, although there was no change in the positive and negative time spent in the forced swim test between these groups (Figure 1C, Supplementary Figure S2). The increased sucrose preference in CUMS mice receiving CSS was abolished in mice receiving both CSS and CHOL, indicating that BAs likely contribute to the antidepressant effect of CSS (*p* < 0.05, Figure 1C). To provide further evidence of a role for BAs in the antidepressant actions of CSS, CUMS mice receiving both CSS + CHOL were supplemented with CA to provide an additional BA source and counteract the effect of CHOL. The sucrose preference index was completely restored in the CSS + CHOL + CA group, with levels similar to control mice (*p* < 0.05). Collectively, these data strongly implicate a critical role for BAs in the antidepressant actions of CSS.

### Chaihu-shugan-san alters the serum bile acid profile of chronic unpredictable mild stress mice

**FIGURE 2:**
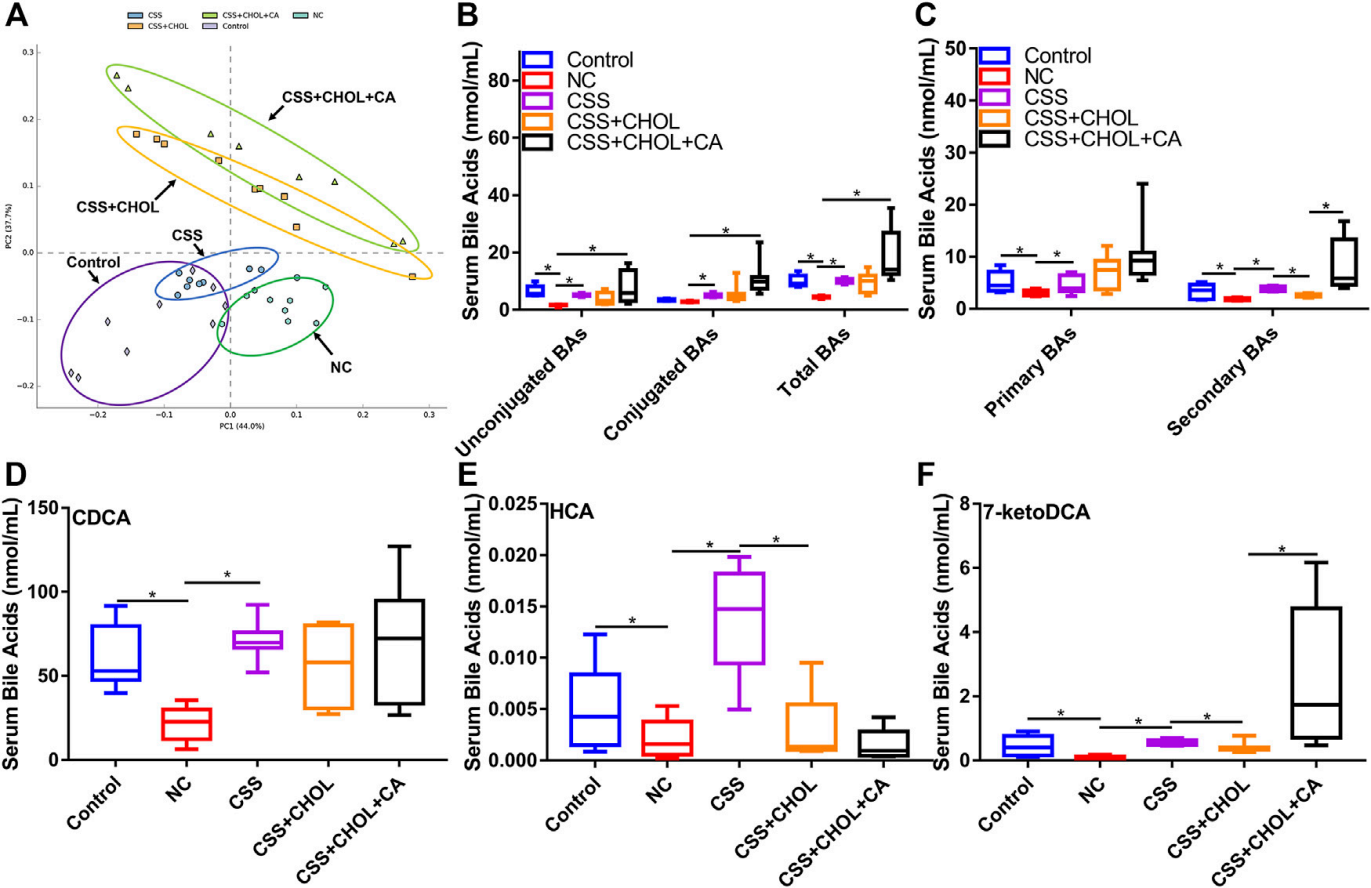
CSS restores serum BA levels in CUMS mice using a mechanism dependent on intestinal reabsorption. MS was used to profile levels of 42 distinct BAs in serum from control mice as well as mice exposed to CUMS and treated with saline (NC), CSS, or CSS along with the BA sequestrant CHOL (with or without CA supplementation). **(A)** PCA plot highlighting different groups based on serum BA concentrations. **(B)** Concentration of unconjugated, conjugated, and total BAs in serum collected from different groups. **(C)** Concentration of primary and secondary BAs in serum isolated from different groups. **(D–F)** Serum levels of **(D)** CDCA **(E)** HCA, and **(F)** 7-ketoDCA in each group. All values are expressed as the mean ± S.D. An asterisk “*” indicates *p* < 0.05 (*n* = 8-9 mice in each group).

To provide insight into changes in serum BA levels between different groups, serum was collected from control, NC, CSS, CSS + CHOL, and CSS + CHOL + CA groups, and UHPLC-QQQ MS analysis was performed to measure levels of 42 distinct BAs. A PCA plot was used to visualize data and cluster groups based on variability (Figure 2A). Notably, the serum BA profile of the NC group was separated from the control group, whereas the serum BA profile of the CSS group had significant overlap with that of the control group. The CSS + CHOL and CSS + CHOL + CA groups were separated from all other groups, likely because of the sequestration of BAs following CHOL administration. The concentrations of unconjugated and total BAs were substantially decreased in the NC group compared with the control group (Figure 2B, *p* < 0.05). Compared with the NC group, the CSS group had higher concentrations of unconjugated, conjugated, and total BAs, with levels similar to the control group (Figure 2B, *p* < 0.05).

CHOL binds and sequesters BAs in the intestine, where biotransformation of primary to secondary BAs by gut microbiota occurs. Sequestration of BAs by CHOL prevents their reabsorption by the intestine into the circulation, so we therefore analyzed serum concentrations of primary and secondary BAs between groups. Compared with the control group, concentrations of primary and secondary BAs in the NC group were robustly decreased, but this effect (particularly for secondary BAs) was mitigated in mice receiving CSS (Figure 2C, *p* < 0.05). Administration of CHOL to mice receiving CSS (CSS + CHOL) did not affect serum levels of primary BAs but significantly decreased secondary BAs to levels similar to the NC group. The effect of CHOL on serum secondary BA levels was circumvented by administering CA (Figure 2C, *p* < 0.05). Therefore, CSS restores BAs in CUMS mice to levels comparable with control mice.

To delineate specific BAs that potentially mediate the antidepressant activity of CSS, we next determined serum levels of individual BAs that were changed following exposure of CUMS mice to CSS. Out of the 42 distinct BAs analyzed in serum, 17 were upregulated following CSS treatment compared to NC mice, with the primary BA CDCA, and secondary BAs HCA and 7-ketoDCA showing the most pronounced differences (Table S11-12). Specifically, compared with the control group, serum CDCA concentration in the NC group was decreased by 65%, but this was completely prevented in mice treated with CSS (Figure 2D, *p* < 0.05). Likewise, compared with the control group, serum levels of HCA and 7-ketoDCA were decreased by 62% and 85% in the NC group, respectively, whereas levels of these BAs in CSS-treated CUMS mice were similar to control mice (Figures 2E,F, *p* < 0.05). Treating mice with CHOL did not affect serum levels of CDCA, but decreased levels of HCA and 7-ketoDCA; the latter effect was prevented by supplementing mice with CA (Figures 2E,F, *p* < 0.05). Overall, CSS increased serum levels of a variety of BAs in CUMS mice, and this effect was modified through sequestration of BAs in the intestine.

### Effect of chaihu-shugan-san and cholestyramine on the liver bile acid profile of chronic unpredictable mild stress mice

**FIGURE 3:**
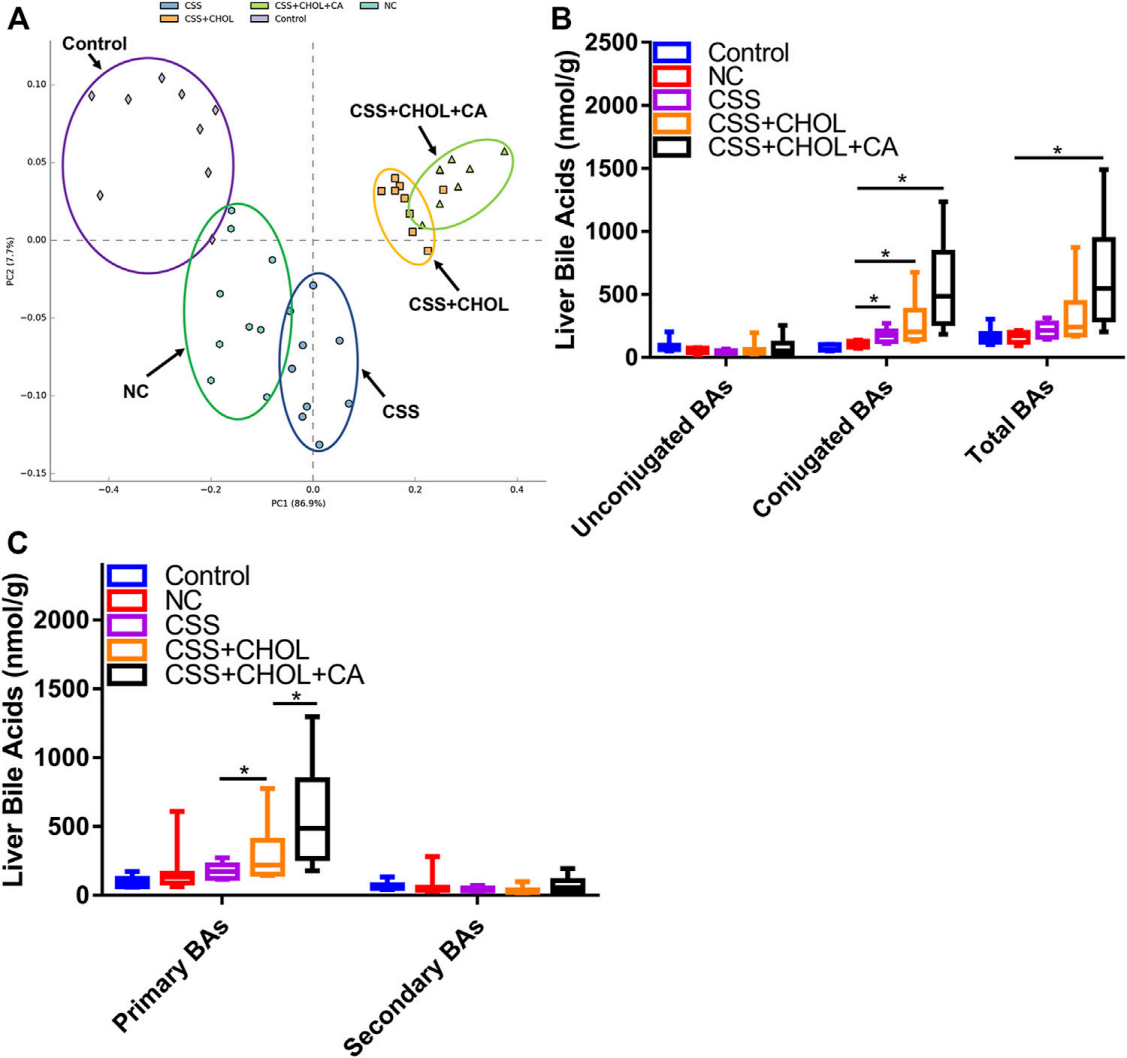
Effect of CUMS and CSS on hepatic BA levels. Levels of 42 distinct BAs were measured in liver tissue collected from mice exposed to CUMS and treated with saline (NC), CSS, or CSS along with the BA sequestrant CHOL (with and without CA supplementation). Mice not exposed to CUMS and treated with saline were used as the control group. **(A)** PCA plot showing different groups based on hepatic BA concentrations. **(B)** Levels of unconjugated, conjugated, and total BAs in livers collected from different groups. **(C)** Levels of primary and secondary BAs in livers from different groups. All values are expressed as means ± S.D. An asterisk “*” indicates *p* < 0.05 (*n* = 8-9 mice per group).

Using a similar strategy as above, we next determined whether exposing mice to the CUMS protocol altered the profile of BAs in the liver, and whether CSS modified this effect. A PCA plot was used to highlight similarities and differences between distinct groups (Figure 3A). The liver BA profiles of the control, NC, CSS CSS + CHOL, and CSS + CHOL + CA groups were separated from each other. In particular, the CHOL-treated mice showed a marked deviation compared to the groups not treated with CHOL.

When determining levels of specific BAs, we found that hepatic concentrations of unconjugated, conjugated, and total BAs (both primary and secondary) remained stable between the control and NC groups, indicating that the CUMS protocol did not affect production or metabolism of BAs in the liver. CSS treatment did not affect levels of unconjugated or total BAs, but elicited a moderate increase in the levels of conjugated BAs (Figures 3B,C, *p* < 0.05). There appeared to be an increase in liver levels of 7-ketoDCA in the CSS group compared to the NC group, but this did not reach statistical significance (*p* = 0.071, Supplementary Table S12). Compared with the CSS group, the CSS + CHOL group had increased levels of conjugated and primary BAs (likely a compensatory response due to reduced intestinal reabsorption of BAs) but no change in secondary or total BAs. Levels of primary BAs were further elevated following supplementation with CA, which was expected since mice were administered CA directly (Figure 3C, *p* < 0.05). Nevertheless, our findings indicate that unlike the robust changes in BA levels in serum following CSS treatment, major changes in liver BA production and metabolism were not apparent. Changes in liver BA levels or metabolism are therefore unlikely to account for the antidepressant actions of CSS.

### Effect of chaihu-shugan-san and bile acid alteration on the gut microbiota profile of chronic unpredictable mild stress mice

Serum levels of secondary BAs such as HCA and 7-ketoDCA were profoundly decreased in mice exposed to the CUMS protocol, whereas treating mice with CSS mitigated this effect. Since secondary BAs are generated by gut microbiota, we next used 16S rRNA sequencing to investigate whether CSS altered the constitution of gut microbiota.

**FIGURE 4:**
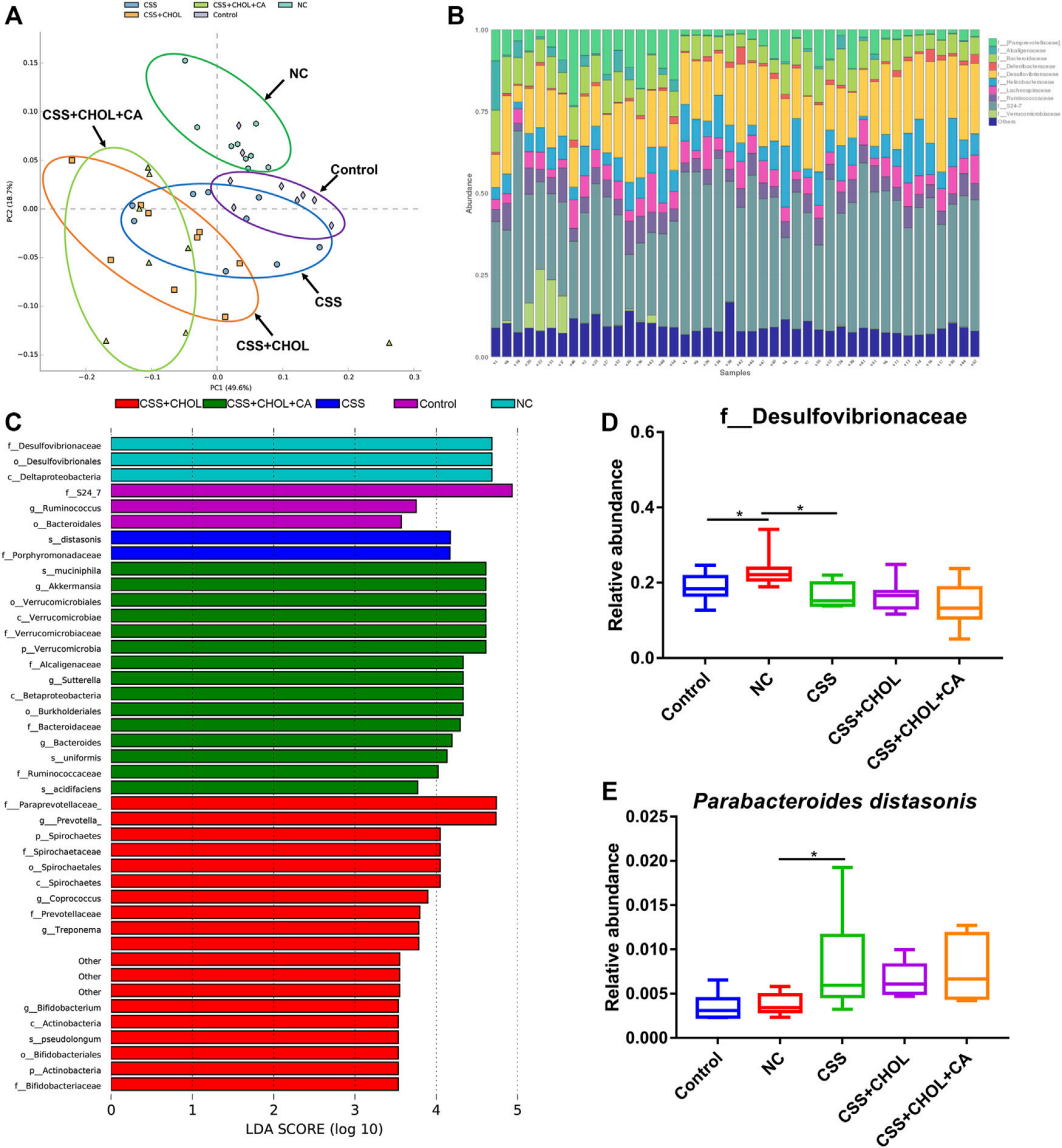
CSS restores the normal intestinal microflora constitution in mice exposed to CUMS. 16S rRNA sequencing was performed using colon contents of control mice as well as mice exposed to CUMS and treated with saline (NC), CSS, or CSS with the BA chelator CHOL (with or without CA). **(A)** PCA plot comparing groups based on abundance of bacterial families within the colon. **(B)** Relative amounts of bacterial families within the colon from each group. **(C)** LDA analysis comparing intestinal microflora between groups. **(D,E)** Relative abundance of **(D)** f_Desulfovibrionaceae and **(E)** *Parabacteroides distasonis* in colons collected from different groups. Values in **(D,E)** are expressed as means ± S.D. An asterisk “*” denotes *p* < 0.05 (*n* = 8-9 mice per group).

PCA analysis depicting the abundance and diversity of gut microbiota in mice from different groups is shown in Figure 4A. The gut microbiota profiles of the control and NC groups were separated from each other, whereas the gut microbiota profile of the CSS group was more similar (and partially overlapped) with the control group. The gut microbiota profiles of the CSS + CHOL and CSS + CHOL + CA groups were similar to each other and partially overlapped with the CSS group.

The relative abundance of the most prominent bacteria families is shown in Figure 4B. We consistently detected ten families within samples, with members of the S24-7 and Desulfovibrionaceae families being most abundant. To determine whether CUMS mice had changes in the composition of intestinal microflora and whether treatment with CSS modified this effect, a linear discriminant analysis (LDA) effect size (LEfSe) analysis was used to detect differences in bacterial abundance. An LDA value greater than 3.5 was considered as the screening standard to compare abundance of microorganisms between groups. As shown in Figure 4C, 42 different OTUs achieved an LDA score greater than 3.5. Bacteria belonging to the Desulfovibrionaceae family were significantly enriched in the NC group compared to the control group, whereas treatment with CSS ameliorated this effect (Figure 4D, *p* < 0.05). Conversely, *P. distasonis* was enriched in CUMS mice receiving CSS, which was not affected by supplementation with CHOL or CA (Figure 4E, *p* < 0.05). Therefore, mice exposed to CUMS have an altered composition of gut bacteria, which is mitigated by treatment with CSS.

### Effect of chaihu-shugan-san on expression of genes associated with bile acid transport and neurotrophic signaling in the hippocampus

**FIGURE 5:**
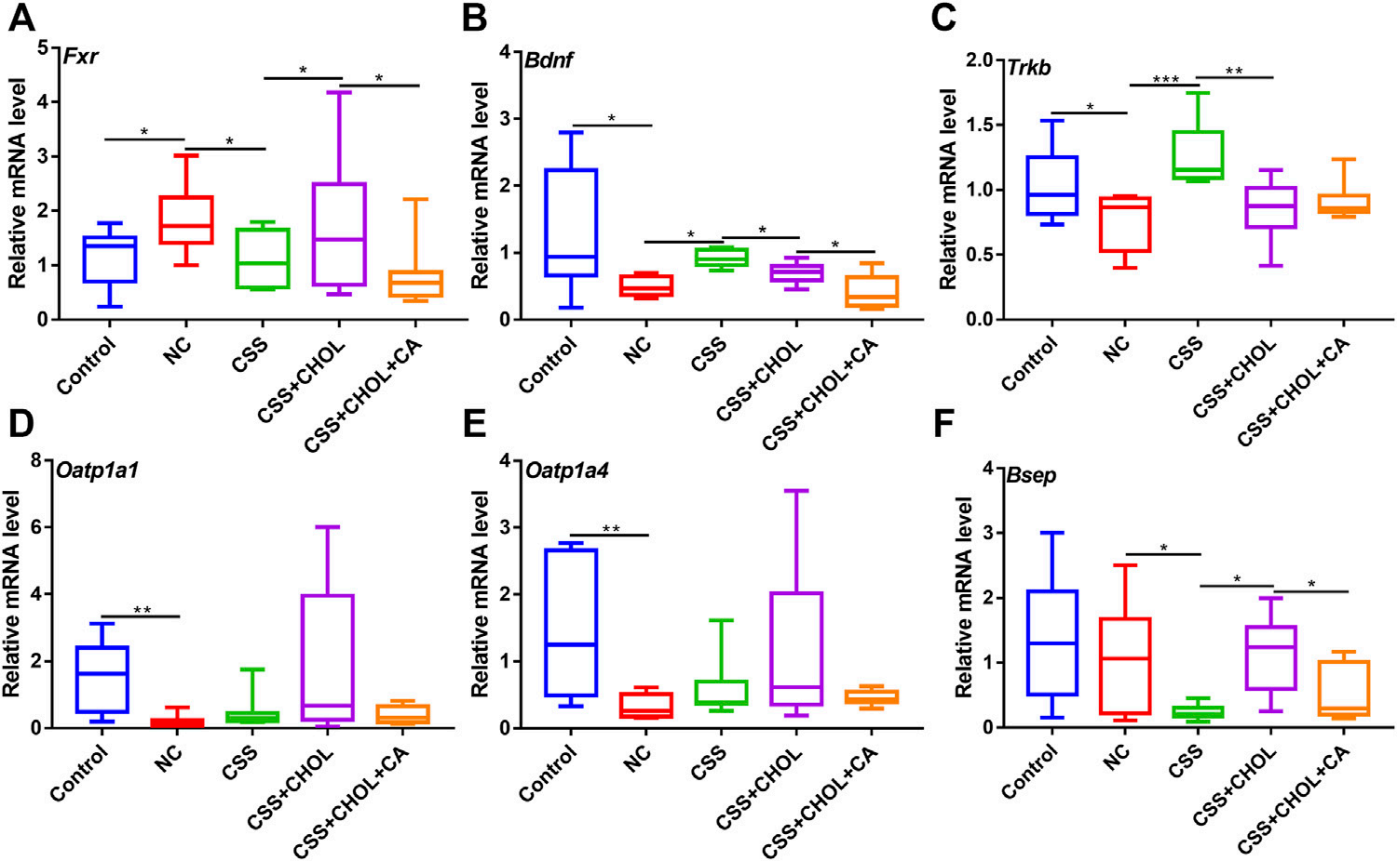
CSS alters expression of genes associated with BA transport and neurotrophic signaling in the hippocampus of CUMS mice. Hippocampi were collected from control mice as well as mice exposed to CUMS and treated with CSS or CSS + CHOL (with or without CA). Transcript levels of **(A)** *Oatp1a1* **(B)** *Oatp1a4*, **(C)** *Bsep* **(D)** *Fxr* **(E)** *Bdnf* and **(F)** *TrkB* were detected by RT-qPCR. Values are expressed as means ± S.D. An asterisk “*” indicates *p* < 0.05, “**” indicates *p* < 0.01, and “***” denotes *p* < 0.001 (*n* = 8-9 mice per group).

Circulating unconjugated BAs can be transported across the blood brain barrier and enter cells within the hippocampus *via* the BA transporters organic anion transporting protein 1a1 (OATP1A1) and OATP1A4, or excreted through the BA efflux transporter, bile salt export pump (BSEP). Since CUMS mice showed signs of depression as well as altered serum BA levels, both of which were mitigated by exposure to CSS, our next goal was to determine whether CUMS mice exhibited altered expression of BA transporters in the hippocampus. As shown in Figure 5, *Oatp1a1* and *Oatp1a4* expression was significantly decreased in the NC group compared with the control group by 86.6% and 77.7%, respectively, whereas there was no change in *Bsep* expression between these groups (*p* < 0.05, Figures 5A–C). Conversely, in CUMS + CSS mice, there was no change in expression of *Oatp1a1* and *Oatp1a4* compared to NC mice but *Bsep* expression was significantly decreased by 77% (*p* < 0.05, Figures 5A–C). Decreased expression of *Bsep* in CSS mice was prevented in mice receiving CHOL, which in turn was overridden by supplementing mice with CA (*p* < 0.05, Figure 5C). These findings indicate that CSS may promote accumulation of BAs in the hippocampus by reducing efflux, and that the effects of CSS on *Bsep* expression are likely mediated by the actions of BA metabolism in the intestine.

BAs act as endogenous ligands of the FXR nuclear transcription factor, and can also directly regulate its expression (Klaassen and Aleksunes, 2010). FXR, in turn, inhibits expression of genes encoding the neurotrophic factors TrkB and BDNF, which are highly implicated in the pathogenesis of depression (Hu et al., 2020). Thus, we next determined whether exposing mice to CUMS altered hippocampal expression of *Fxr*, *TrkB*, and *Bdnf*, and whether CSS treatment restored hippocampal expression of these genes. Expression of *Fxr* was increased in the NC group compared with the control group, and this was inhibited by treating mice with CSS. Administration of CHOL + CSS increased expression of *Fxr* to levels consistent with the NC group, and this was prevented by supplementing mice with CA (Figure 5D, *p* < 0.05). Thus, CSS decreases *Fxr* expression through a mechanism that likely involves BAs. The increased expression of *Fxr* in the NC group was associated with reduced expression of *Bdnf* and *TrkB*, whereas relatively higher expression of these neurotrophic factors was evident in mice treated with CSS, with levels similar to control mice (*p* < 0.05, Figures 5E,F). Mice receiving CSS + CHOL had decreased expression of *Bdnf* and *TrkB* similar to the NC group, although CA supplementation did not restore expression of these genes. Thus, mice exposed to the CUMS protocol to elicit depression-like symptoms have altered expression of BA transporters and signaling mediators as well as neurotrophic factors in the hippocampus, and expression of a subset of these factors is restored to control levels following CSS treatment through a BA-mediated mechanism.

### Gut microbiota from chaihu-shugan-san mice alleviate depression-like symptoms in chronic unpredictable mild stress mice

Since we identified increased levels of secondary BAs (i.e., HCA and 7-ketoDCA) in serum following treatment with CSS, and this was associated with altered levels of *P. distasonis* and *Desulfovibrio* in the colon, we next performed a correlation analysis comparing the concentration of serum BAs with the relative abundance of distinct gut microbiota. Spearman correlation analysis revealed that differential serum levels of HCA were positively correlated with the abundance of *Parabacteroides* (*p* < 0.05, Supplementary Figure S3). Therefore, we postulated that CSS may alleviate depression-like symptoms in CUMS mice, at least in part, by modulating the abundance of BA-transforming gut microbiota, resulting in altered production and reabsorption of secondary BAs.

To determine whether gut bacteria are sufficient to exert antidepressant effects in CUMS mice, an FMT rescue strategy was adopted wherein bacteria from CSS-treated mice were transplanted into untreated CUMS mice. An additional control group was included in which fecal material was filtered through a 0.22 μm membrane before transplantation (filtered FMT, FFMT) to evaluate the potential of the transplantation procedure and fecal material other than microbiota to exert an effect.

**FIGURE 6:**
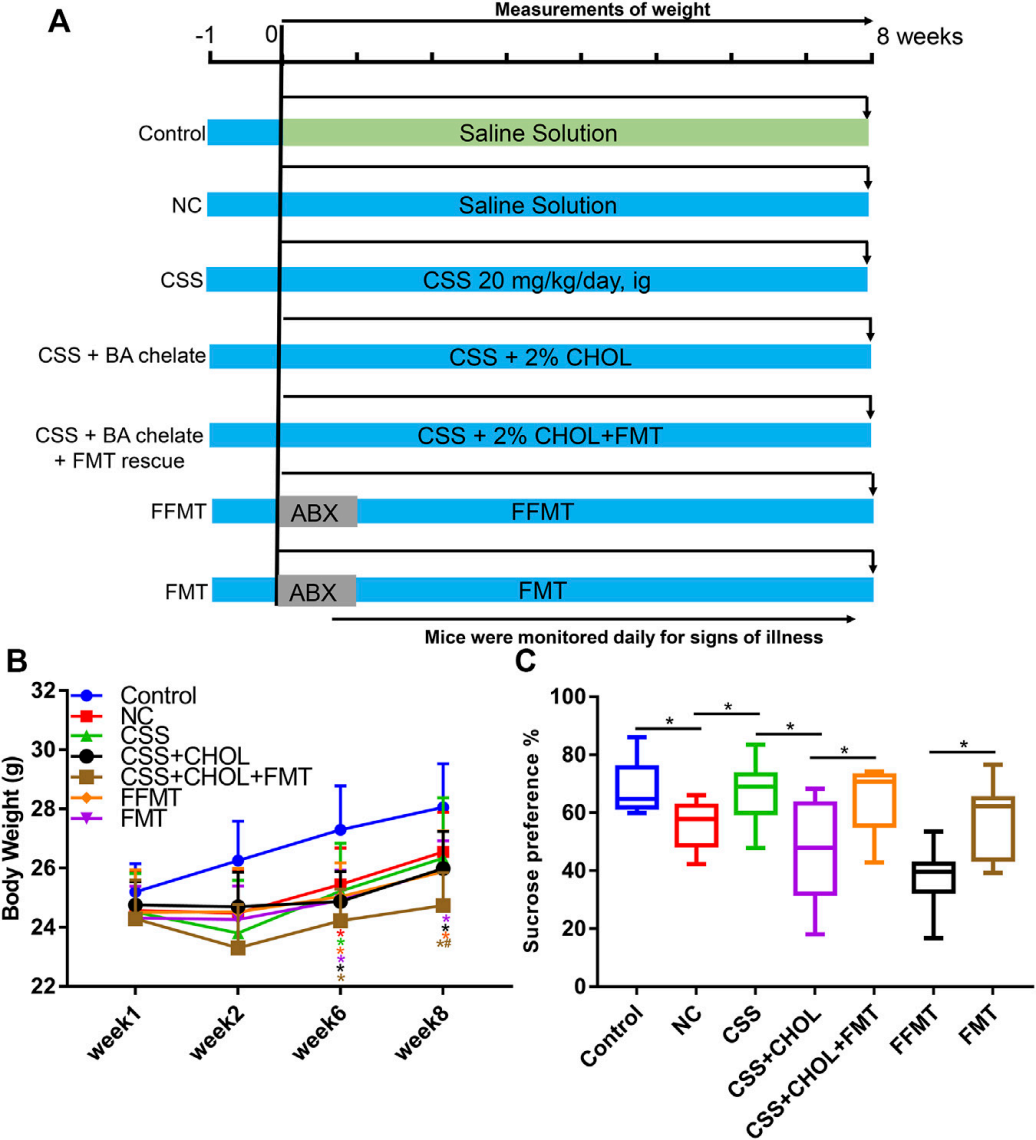
Transplantation of microbiota from CSS-treated mice into untreated mice alleviates depression-like symptoms after exposure to CUMS. Mice were exposed to CUMS and treated with saline (NC group), CSS, or CSS along with the BA sequestrant CHOL. Control mice received saline but were not exposed to CUMS. Additional groups of CUMS mice were treated with antibiotics (ABX) and then received fecal microbiota from CSS-treated groups (FMT) or filtered FMT (FFMT) to remove microbiota. **(A)** Overview of experimental groups and timelines. **(B)** Body weights of mice measured 1, 2, 6, and 8 weeks after initiating the experiment. **(C)** Percent sucrose preference from each group (based on consumption of 1% sucrose in the sucrose preference test). All values are expressed as the mean ± S.D. An asterisk “*” indicates *p* < 0.05 (*n* = 7-8 mice per group).

Compared with the control group, the body weights of all groups of mice exposed to the CUMS protocol were decreased at 6 and 8 weeks regardless of treatment (*p* < 0.05, Figure 6B). The sucrose preference index and positive time in the forced swim test were decreased in the NC group compared to the control group, whereas immobility time was significantly increased, confirming that the CUMS protocol elicited depression-like symptoms in these mice (*p* < 0.05, Figure 6C, Supplementary Figure S4). Consistent with our previous observations, depression-like symptoms were mitigated in CSS-treated mice such that their sucrose preference index and results in the forced swim test were similar to controls, and the antidepressant actions of CSS were prevented by sequestering BAs in the intestine using CHOL (*p* < 0.05, Figure 6C, Supplementary Figure S4). Mice exposed to FFMT exhibited a substantial decrease in sucrose preference index compared to both control and NC groups, which may relate to the transplantation procedure. However, in forced swim tests, FFMT mice behaved similarly to the NC group (*p* < 0.05, Figure 6C, Supplementary Figure S4). Compared with the FFMT group, mice exposed to FMT (with or without CHOL) showed an increased sucrose preference (*p* < 0.05, Figure 6C). Mice exposed to FMT also had increased positive time and decreased immobility time in the forced swim test compared to the FFMT group, suggesting that transplantation of gut bacteria from CSS-treated mice is sufficient to reduce depression-like symptoms (*p* < 0.05, Supplementary Figure S4).

### Gut microbiota altered by chaihu-shugan-san affects the serum bile acid profile of chronic unpredictable mild stress mice

**FIGURE 7:**
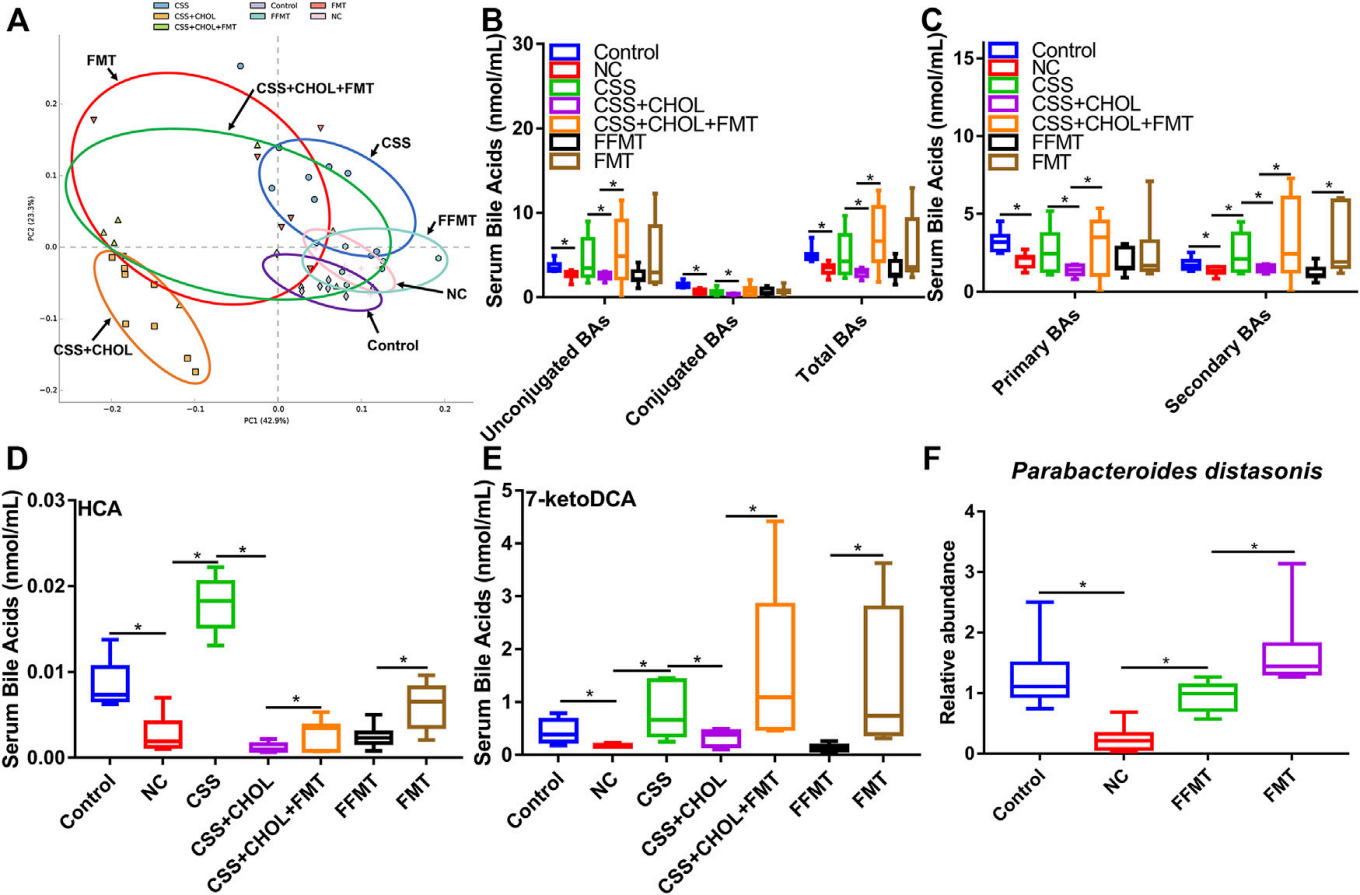
Transplantation of microbiota from CSS-treated mice into untreated mice restores serum BA levels in CUMS mice. MS was used to measure levels of BAs in serum from control mice as well as mice exposed to CUMS and treated with saline (NC), CSS, or CSS along with the BA sequestrant CHOL; or fecal contents (unfiltered or filtered) from CSS-treated mice (FMT and FFMT, respectively). **(A)** PCA plot comparing serum BA concentrations between groups. **(B)** Levels of unconjugated, conjugated and total BAs in serum collected from different groups. **(C)** Levels of primary and secondary BAs in serum collected from different groups. **(D,E)** Serum levels of **(D)** HCA, and **(E)** 7-ketoDCA in each group. **(F)** Relative abundance of *Parabacteroides distasonis* (detected by RT-qPCR) in colon contents of control mice or mice exposed to CUMS and treated with saline, FMT, or FFMT. All values are expressed as means ± S.D. An asterisk “*” indicates *p* < 0.05.

We next determined whether FMT from CSS-treated mice transplanted into untreated mice was sufficient to alter serum BA profiles. PCA analysis of serum BAs of mice from different experimental groups is shown in Figure 7A. The serum BA profiles of the NC, CSS, FMT, CSS + CHOL, and CSS + CHOL + FMT groups were separated from that of the control group. FFMT was separated from FMT and showed a BA profile similar to that of the NC group. The serum BA profile of the CSS + CHOL + FMT group was similar to that of the FMT group.

Serum concentrations of total BAs (including both unconjugated and conjugated) were decreased in the NC group compared to the control group (Figure 7B, *p* < 0.05). Likewise, levels of both primary and secondary BAs were decreased in the NC group. Treatment with CSS restored secondary BAs to levels similar to the control group, and this effect was blocked in CSS + CHOL mice (Figure 7C, *p* < 0.05). Intriguingly, FMT (with or without CHOL), but not FFMT, was sufficient to increase secondary BAs to levels consistent with those detected in CSS-treated mice (Figure 7C, *p* < 0.05), indicating that CSS-induced changes in the gut microbiota are mechanistically involved in elevating the serum profile of secondary BAs.

**FIGURE 8:**
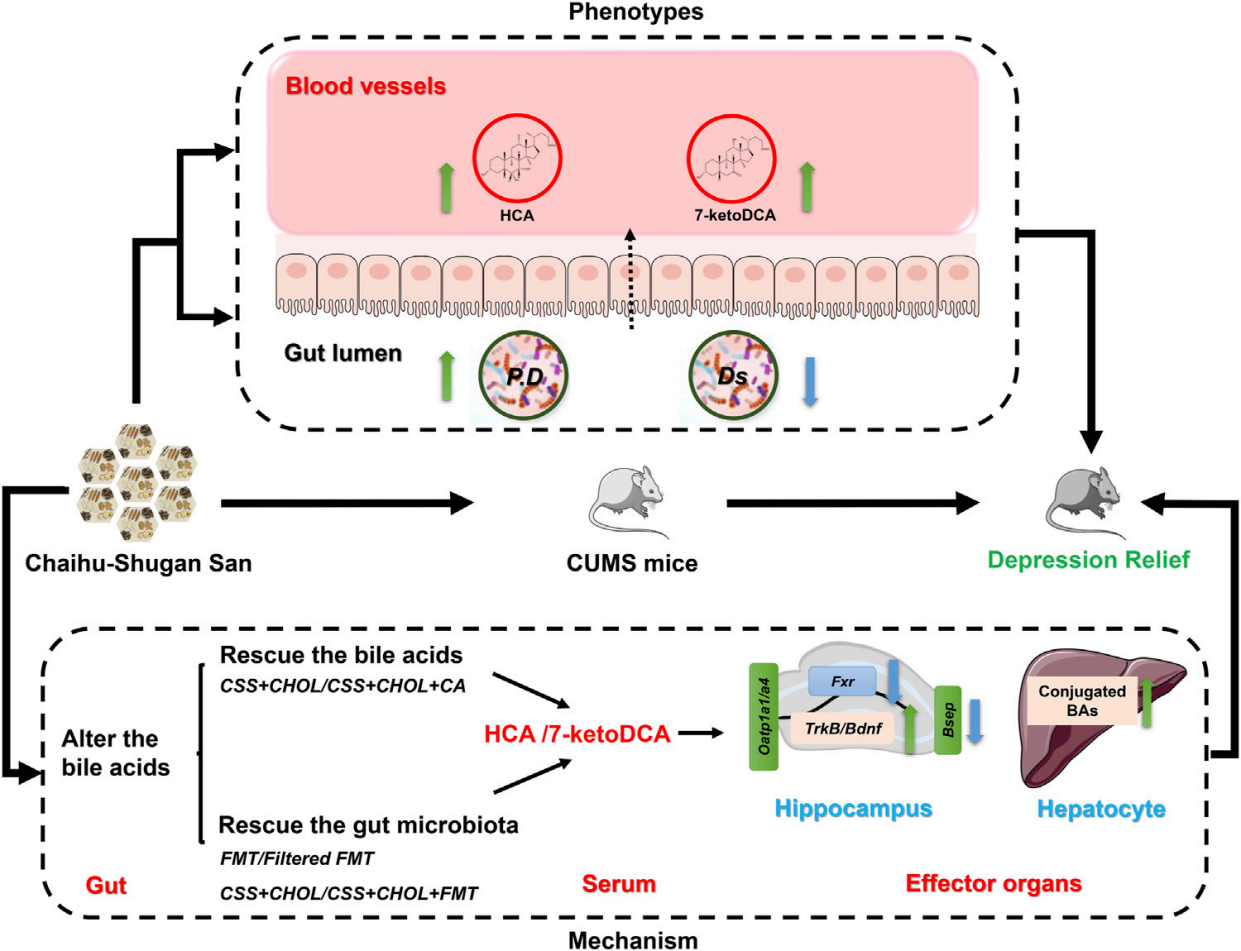
Overview of potential mechanism of CSS antidepressant action. We propose that CSS mitigates depression in CUMS mice by altering the constitution of gut microflora, thereby increasing serum levels of specific BAs and metabolites such as HCA and 7-ketoDCA. These BAs regulate critical cell signaling cascades in the hippocampus such as neurotrophins, which are implicated in the pathogenesis of depression. P. D: *Parabacteriods distasonis*; Ds: *Desulfovibrio.*

Since HCA and 7-ketoDCA were identified in our previous analyses as secondary BAs that showed pronounced changes following CSS treatment, we next assessed serum levels of these BAs following transplantation of fecal material from CSS-treated mice into untreated mice. Similar to our previous observations, serum levels of HCA and 7-ketoDCA were robustly decreased in the NC group compared to the control group, which correlated with an 80.6% decrease in the intestinal abundance *of P. distasonis* (Figures 7D–F; Supplementary Table S11). Serum levels of HCA and 7-ketoDCA were restored in mice treated with CSS, and this effect was inhibited by sequestering BAs in the intestine using CHOL. FMT had a moderate but significant increase in serum HCA levels compared to FFMT, and showed a prominent increase (both with and without CHOL) in serum 7-ketoDCA levels comparable or higher than both the control and CSS groups (*p* < 0.05, Figures 7D,E). Although the FFMT group showed a slight but significant increase in intestinal *P. distasonis* abundance compared to the NC group, the FMT group had the most pronounced increase in the abundance of this bacterial species, and there was a significant correlation between colonic *P. distasonis* abundance and serum concentration of 7-ketoDCA (*p* < 0.05, Figure 7F and Supplementary Figure S5)*.* Thus, we propose that CSS mediates its antidepressant actions by altering the constituency of the gut microflora, resulting in increased levels of specific BAs in serum (e.g., HCA and 7-ketoDCA) capable of regulating cellular signaling events in the hippocampus, such as BA transport and neurotrophin signaling (Figure 8).

## Discussion

CSS is a classical antidepressant formula for clinical application particularly in China, yet its mechanism of action remains unclear. There is now substantial evidence that the microbiota-gut-brain axis is critical for normal brain functions. BAs and other metabolites are synthesized and transformed by the intestinal flora, reabsorbed *via* the portal circulation, and enter into various tissues including the brain to exert pleiotropic actions. In this study, we found that reduced levels of specific BAs are associated with CUMS-induced depression in mice, and that CSS mitigates symptoms of depression by altering the constitution of gut microflora and restoring serum BA levels. Our findings provide new insight into the mechanism through which CSS exerts antidepressant actions, and lend support to the notion that the microbiota-gut-brain axis represents an intriguing therapeutic target for ameliorating serious neurological disorders like depression.

Previous non-targeted metabolomics studies using rats have found that CSS influences BA metabolism (Su et al., 2014; Jia et al., 2017); however, the role of BAs in the antidepressant effect of CSS has not yet been reported. In this study, we used an established and reliable animal model of depression in which mice are exposed to CUMS, and found that serum levels of secondary BAs–HCA and 7-ketoDCA–were decreased in these mice likely due to altered intestinal microflora constitution. Treatment of these mice with CSS restored intestinal microflora constitution and serum BAs to levels comparable to control mice, correlating with reduced depression-like symptoms. Sequestration of BAs in the intestine (using CHOL) inhibited the effects of CSS on serum BA levels, expression of key genes associated with neurotrophin signaling in the hippocampus, and behavior; whereas administration of CA was able to reverse most effects of CHOL. Furthermore, treatment of CUMS mice with unfiltered fecal material (containing microbiota) from CSS-treated mice was sufficient to increase BA levels and obviate depression-like symptoms. Therefore, our findings strongly suggest that BAs synthesized by intestinal microflora have a critical role in facilitating the antidepressant actions of CSS.

The sucrose preference test and forced swim test are classical behavioral experiments for evaluating the level of anhedonia and desperation, and are thus used as indicators of depression-like symptoms in animal models. In the present study, mice exposed to CUMS showed decreased preference for sucrose, and this was alleviated by CSS (Figure 1C), which is consistent with another study using a distinct mouse model of depression (Li et al., 2019). However, unlike this other study, CSS did not consistently alter immobility time in the forced swim test (Supplementary Figures S2A,B), which may be due to differences in experimental approaches and the dose of CSS administered (He et al., 2019). Anhedonia is typically considered an early symptom of depression, whereas patients with more severe depression (like major depressive disorder) are more likely to exhibit signs of desperation. Therefore, a low dose of CSS could be effective in patients showing early signs of depression such as anhedonia, but higher doses or alternative approaches may be needed to treat depression in more severe cases.

Our findings indicate that serum BAs play a pivotal role in the antidepressant effect of CSS. In particular, serum levels of HCA and 7-ketoDCA were strongly associated with the antidepressant effect of CSS; serum levels of HCA and 7-ketoDCA were profoundly affected in both the BA sequestration and FMT rescue experiments (Figures 2, 7). HCA is converted from CDCA by 6-hydroxylation, while 7-ketoDCA is derived from CA following metabolism by intestinal bacteria such as *P. distasonis* (Spinelli et al., 2016; Wang et al., 2019). Interestingly, CSS also increased the serum concentration of CA and CDCA in CUMS mice. It is therefore possible that CSS increases serum levels of 7-ketoDCA and HCA indirectly by inducing levels of their precursor BAs. Nevertheless, the secondary BAs HCA and 7-ketoDCA appear to be functionally involved in mediating the antidepressant effects of CSS.

Multiple studies have emphasized the relationship between gut microflora constitution and depression (Yu et al., 2019; Yang et al., 2020a). Thus, CSS may alleviate depression-like symptoms in CUMS mice by reestablishing the gut microflora composition to one more akin to control mice. Our study aligns with this premise since CSS shifted the gut microbiota profile from CUMS mice to one more similar to control mice, suggesting that colon bacteria may be involved in the antidepressant effect of CSS. To further investigate the potential role of intestinal microflora in the antidepressant actions of CSS, we used our patented FMT procedure (ZL 2018 1 061243.4), which is designed to determine how the intestinal microflora influences the actions and efficacy of drugs. By transplanting intestinal bacteria from CSS-treated mice into untreated CUMS mice, we demonstrated that intestinal microflora contributes to CSS-induced restoration of serum BA levels and improved behavioral outcomes in CUMS mice.

When analyzing the gut microflora composition, decreased abundance of *P. distasonis* and increased Desulfovibrionaceae family members were apparent in CUMS mice compared to controls. Treatment of CUMS mice with CSS restored the composition of the intestinal flora similar to controls, suggesting that these particular bacteria may contribute to the altered BA levels and antidepressant actions induced by CSS. Desulfovibrionacea encompasses a family of anaerobic Gram-negative bacteria, and they have been associated with inflammation and disruption of the gut-microbiota-brain axis in part through production of H_2_S (Yang et al., 2017; Zhu et al., 2019). Consistent with our study, increased Desulfovibrionacea abundance in mice has been associated with neurological disorders including restraint-induced depression as well as symptoms reminiscent of Alzheimer’s disease and Parkinson’s disease (Han et al., 2021; Murros et al., 2021; Zhao et al., 2021; Yang et al., 2022; Zhu et al., 2022). *P. distasonis*, on the other hand, is rich in BA metabolizing enzymes, including bile salt hydrolase and 7-α hydroxyoxidase, and can efficiently transform primary BAs into secondary BAs such as conversion of CA to 7-ketoDCA (Wang et al., 2019). Thus, increased levels of secondary BAs resulting in mitigation of depression-like symptoms following CSS treatment in CUMS mice may be due, at least in part, to greater abundance of *P. distasonis*.

Recently, oral administration of *P. distasonis* was reported to inhibit depressive behavior of mice undergoing chronic restraint by increasing the level of 5-HT in the hippocampus and inhibiting the kynurenine metabolic pathway (Deng et al., 2021). Conversely, *P. distasonis* was shown to exacerbate depressive behavior in a mouse model of Crohn’s disease (Gomez-Nguyen et al., 2021), indicating that the relationship between *P. distasonis* and behavior may be context-specific. It is also important to investigate the composition of the intestinal microbiota *en masse* rather than focusing on a single bacterial species, since changes in other microbial families were noteworthy. For instance, *Bifidobacterium* and *Treponema* were enriched in the CHOL group, whereas *Akkermansia* and *Bacteroides* were more abundant in the CHOL group receiving CA supplementation. *Treponema* (e.g., *T. pallidum* and *T. pedis*) are often present in pathogenic responses, including syphilis and inflammation (Arrazuria et al., 2020; Lu et al., 2020). Oral administration of *Bifidobacterium longum* R0175, *B. bifidum* W23 and *B. lactis* W52 alleviates depressive behavior in humans and rodent models (Kudchodkar et al., 1977; Tian et al., 2019; Han et al., 2020). In a mouse model of depression, abundance of *Akkermansia* was decreased whereas *Bacteroides* was enriched; but administration of *B. fragilis* alleviated anxiety behavior in mice (McGaughey et al., 2019; Yan et al., 2020b; Yang et al., 2020b). Thus, although CSS treatment was associated with higher abundance of *P. distasonis* and this correlated with reduced symptoms of depression in CUMS mice, changes to the intestinal flora in response to various treatments is complex. The resulting effect on serum BA levels and behavior is likely due to the confluence of changes to the composition of the intestinal flora, which may explain some of the variability noted in our study.

The transport of BAs is regulated through BA transporters such as OATP1A1, OATP1A4, and BSEP. Since BAs likely facilitate the potent antidepressant actions of CSS, BA transporters and signaling cascades may be interesting therapeutic targets for the treatment of depression. Expression of *Oatp1a1* and *Oatp1a4* (which encode proteins that uptake unconjugated BAs) were decreased in the hippocampus of CUMS mice. Although CSS treatment did not appear to affect expression of these genes, expression of *Bsep–*encoding the BA efflux transporter–was decreased in the hippocampus following CSS treatment. Therefore, although we were not able to measure BA levels in the hippocampus in this study, we postulate that CSS treatment may restore hippocampal BA levels in CUMS mice by preventing their efflux.

BAs serve as ligands for the G-protein coupled cell surface receptor TGR5 and nuclear receptor FXR. Activation of FXR and TGR5 in the hippocampus has been implicated in neurological disorders. In general, inhibition of FXR and activation of TGR5 in the hippocampus induces BDNF levels and mitigates depression-like symptoms in mice (Huang et al., 2015; Chen et al., 2018b; Hu et al., 2020). Interestingly, HCA was reported to be an antagonist of FXR and an agonist of TGR5 (Zheng et al., 2021). Increased levels of HCA in CSS-treated mice may therefore contribute to decreased signaling through FXR, resulting in elevated levels of *Bdnf* and *TrkB*. To the best of our knowledge, the relationship between 7-ketoDCA and activation of FXR or TGR5 has not been reported. In the present study, we detected increased *Fxr* expression in the hippocampus of CUMS mice along with reduced expression of *Bdnf* and *TrkB*, all of which were reversed by treating mice with CSS. Therefore, the antidepressant actions of CSS may operate, at least in part, through regulating BA availability and signaling networks in the hippocampus and promoting neurotrophin signaling. If this hypothesis is correct, promotion of secondary BA accumulation and signaling in the hippocampus may be a potential therapeutic option to treat depression.

## Data Availability

The datasets presented in this study can be found in online repositories. The names of the repository/repositories and accession number(s) can be found in the article/Supplementary Material.

## Glossary

16S rRNA: 16S ribosomal RNA
3-oxoCA: 3-oxo-cholic acid
6-ketoLCA: 6-ketolitocholic acid
7-ketoDCA: 7-ketodeoxycholic acid
7-ketoLCA: 7-ketolitocholic acid
ACA: Allocholic acid
apoCA: Apocholic acid
BA: Bile acid
BDNF: Brain derived neurotrophic factor
Bsep: bile salt export pump
CA: Cholic acid
CDCA: Chenodeoxycholic acid
CHOL: Cholestyramine
CSS: Chaihu-Shugan San
CUMS: Chronic unpredictable mild stress
D4-CA: D4-Cholic acid
D4-GCA: D4-Glycocholic acid
D4-LCA: D4-Litocholic acid
D4-TCA: D4-Taurocholic acid
DCA: Deoxycholic acid
DHCA: Dihydroxycholestanoic acid
DHLCA: Dehydrolitocholic acid
GCA: Glycocholic acid
FFMT: Filter fecal microbiota transplantation
SPT: Sucrose preference test
FMT: Fecal microbiota transplantation
GCDCA: Glycochenodeoxycholic acid
GDCA: Glycodeoxycholic acid
GDHCA: Glycodihydroxycholestanoic acid
GHCA: Glycohyocholic acid
GHDCA: Glycohyodeoxycholic acid
GLCA: Glycolitocholic acid
GLCA-3S: 3-sulfoglycolitocholic acid
GUDCA: Glycoursodeoxycholic acid
HCA: Hyocholic acid
HDCA: Hyodeoxycholic acid
HQC: High quality control
IS: Internal standard
isoalloLCA: Iso-allolitocholic acid
isoLCA: Iso-litocholic acid
Oatp1a1: Organic anion transporting protein 1a1
Oatp1a4: Organic anion transporting protein 1a4
Trkb: Tyrosine Kinase receptor B
