# Supplementary material for: Chaihu-shugan-san alleviates depression-like behavior in mice exposed to chronic unpredictable stress by altering the gut microbiota and levels of the bile acids hyocholic acid and 7-ketoDCA
Source: Front Pharmacol. 2022 Oct 19;13:1040591. doi: 10.3389/fphar.2022.1040591 (PMC9627339; doi:10.3389/fphar.2022.1040591)
Supplement: Supplementary file 1 [file Table1.DOCX]

**Supplemental Material**

**Chaihu-Shugan-San alleviates depression-like behavior in mice exposed to chronic unpredictable stress by altering the gut microbiota and levels of the bile acids HCA and 7-ketoDCA**

Chong Ma^1,2†^, Yuan Dun^3†^, Stephen James Renaud^4^, Ting Zhou^1^, Fan Yang^1^, Yuligh Liou^5^, Xinjian Qiu^6^, Lu Zhou^6^, and Ying Guo^1*^

*^1^ Department of Clinical Pharmacology,* *Xiangya Hospital, Central South University, 87 Xiangya Road, Changsha 410008, P. R. China; Institute of Clinical Pharmacology, Central South University, Hunan Key Laboratory of Pharmacogenetics, 110 Xiangya Road, Changsha 410078, P. R. China; Engineering Research Center of Applied Technology of Pharmacogenomics, Ministry of Education, 110 Xiangya Road, Changsha 410078, P. R. China, National Clinical Research Center for Geriatric Disorders, Xiangya Hospital, Central South University, Changsha, Hunan, P.R. China (C.M., Y.G.)*

*^2^ School of Pharmaceutical Sciences (Shenzhen), Sun Yat-sen University, Guangzhou, 510006, P.R. China.*

*^3^ Department of Neurosurgery, Xiangya Hospital, Central South University, Changsha, Hunan 410008, P.R. China.*

*^4^ Department of Anatomy and Cell Biology, The University of Western Ontario, London, Ontario, Canada.*

*^5^* *China Xiangya Medical Laboratory, Central South University, Changsha, Hunan, 410078, P.R. China.*

*^6^ Department of Integrated Traditional Chinese and Western Medicine, Xiangya Hospital, Central South University, Changsha.*

†These authors contributed equally to this work and share first authorship

**Detection of the Chaihu-Shugan-San Components Utilizing Ultra-Performance Liquid Chromatography.**

The measurement of UPLC chromatographic was conducted while employing a Waters ACQUITY UPLC series supplied with a quaternary pump, a diode array detector (PDA), an online degasser, and an autosampler controlled by Empower2. For chromatographic separation, Waters BEH C18 column (2.1 × 50 mm, 1.7 μm) was utilized. The mobile phase included acetonitrile (A) and acetic acid (pH 3.5) (B). The curves of gradient elution were: 0–10 min, 5%A: 95%B; 10–20 min, 15%A: 85%B; 20–30 min, 30%A: 70%B; 30–40 min, 50%A: 50%B; 40–45 min, 70%A: 30%B; 45–50 min, 80%A: 20%B. The PDA was set at 190–480 nm. The parameters were set as follows: Column temperature: 40°C; Injection volume: 3 ul; Flow rate: 0.5 ml/ min.

Note: Reference to previous publications of our group. Front Pharmacol. 2022 Jan 17;12:791097. doi: 10.3389/fphar.2021.791097.

**Sample preparation for UHPLC-QQQ MS analysis.**

***Serum Sample Preparation.*** D_4_-LCA, D_4_-CA, D_4_-GCA, and D_4_-TCA were used as internal standards. One milliliter of precooled acetonitrile was added to 50 μL serum spiked with 10 μL of the internal standards (400 ng/mL), vortexed, and centrifuged at 14,000 × g for 15 min. The supernatant was collected and vacuum-dried. The residue was reconstituted in 50 μL of 50% methanol.

***Liver Sample Preparation.*** Approximately 115 ± 5 mg of liver tissue with 10 µL of internal standard (4 μg/mL) was homogenized in 5 volumes of ddH_2_O. Three milliliters of precooled acetonitrile were added to 600 μL of liver homogenate with internal standards, vortexed, and centrifuged at 14,000 × g for 15 min. The supernatant was collected and vacuum-dried. The residue was reconstituted in 500 μL of 50% methanol and then filtered through a 0.22 μm membrane.

**UHPLC–MS/MS analysis of BAs.**

UHPLC-QQQ MS analysis was performed on a Nexera X2 LC-30AD liquid chromatogram (Shimadzu, Japan) and a triple-quadrupole mass spectrometer (QQQ MS 6500+, AB SCIEX, Foster City, USA) equipped with an ACQUITY UPLC HSS T3 (2.1×100 mm, 1.8 μm, Waters, Milford, MA, USA). Mobile phases contained 20% acetonitrile/aqueous solution (A) and 80% acetonitrile/aqueous solution (B). Both mobile phases contained 10 mM ammonium acetate.

The gradient conditions were as follows: 0-1 min, linear gradient from 5% mobile phase B to 8% mobile phase B; 1-10 min, 8% mobile phase B to 14% mobile phase B; 10-11 min, 14% mobile phase B to 25% mobile phase B; 11-15 min, 25% mobile phase B to 25% mobile phase B; 15-16 min, 25% mobile phase B to 50% mobile phase B; 16-19 min, 50% mobile phase B to 50% mobile phase B; 19-22 min, 50% mobile phase B to 95% mobile phase B; 22-24 min, 95% mobile phase B to 95% mobile phase B; 24-24.5 min, 95% mobile phase B to 5% mobile phase B; and 24.5-26 min, 5% mobile phase B to 5% mobile phase B. The flow rate was 0.3 mL/min. The temperatures of the column and the autosampler were set at 40 and 4 ℃, respectively. Mass spectrometry was performed in negative polarity using a capillary voltage of 4.5 kV. The parameters of the instrument were set as follows: GAS1: 50 psi; GAS2: 60 psi; TEM: 550 ℃; CUR: 30 psi. Analytes were detected using a multiple reaction monitor using the auto dwell time function. Additional details are provided in Table S1 and S2.

**Validation of UHPLC–MS/MS.**

Method validation studies were designed by following the FDA’s Guidance for Industry for Bioanalytical Method Validation for testing the limit of detection (LOD) and the lower limit of quantification (LLOQ), linearity, intra- and inter-assay precision, accuracy, matrix effects, and extraction recovery. Identical validation experiments were conducted for serum and liver tissue. The MS data were acquired and processed with Analyst software (version 1.7.1, AB SCIEX, USA). The concentrations of working solutions and QC are shown in Table S3. For the LOD/LLOQ and linearity, BA standard solutions at 7 concentrations containing 4 mixed internal standards were prepared in stripped matrices and injected into the LC in order from low to high concentrations. BA working solutions and QC concentrations are detailed in Table S4.

For the intra- and inter-assay precision and accuracy in serum and liver, 4 mixed BA QCs LLOQ, LQC, MQC, and HQC (Table S3) were analyzed to evaluate the intra- and inter-assay precision and accuracy of the method by testing 6 replicates of each of the 3 consecutive batches. For the extraction recovery and matrix effect of serum and liver, 3 mixed BA QC samples, LQC, MQC, and HQC (Table S1), were analyzed by testing 6 replicate batches. The RSD (%) and accuracy (%) met the quantitative requirements (Table S5-8).

**Fig. S1. A method for studying intestinal microbiota based on fecal filtration and transplantation.**

**
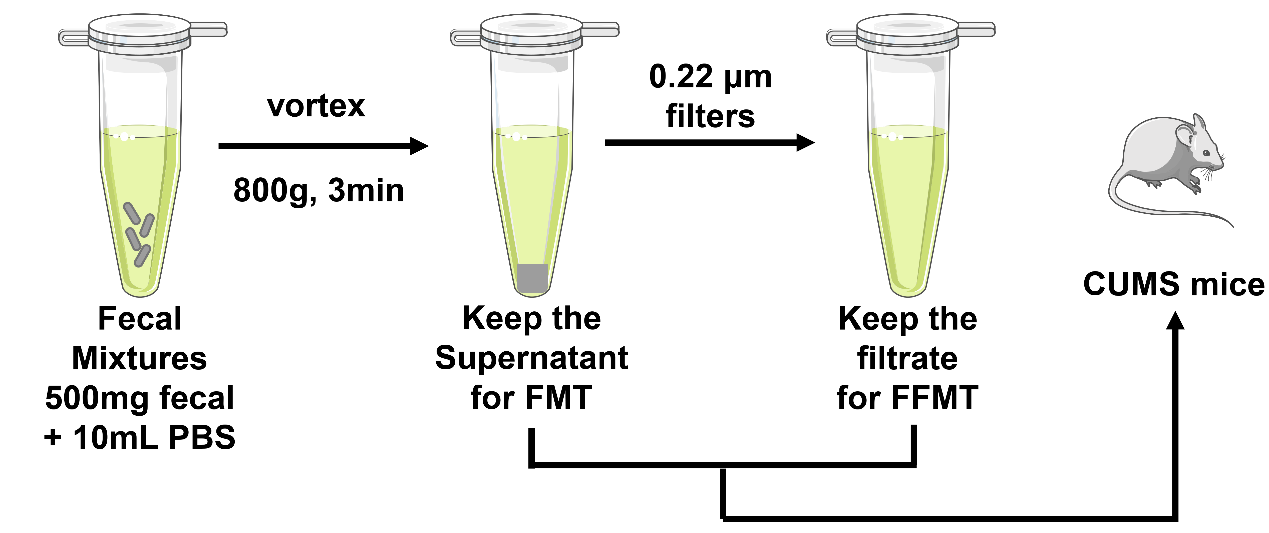
**

**Fig. S2. Effect of CSS and BA manipulation on forced swim test outcomes using a CUMS mouse model of depression.** Mice were exposed to CUMS and treated with saline (NC group), CSS, or CSS along with the BA sequestrant CHOL with or without CA supplementation. Control mice were not exposed to CUMS but received saline. Mice were placed in the forced swim test and the time spent struggling (A; positive time) or immobile (B; negative time) was recorded. All values are expressed as mean ± S.D. An asterisk “*” indicates P<0.05.


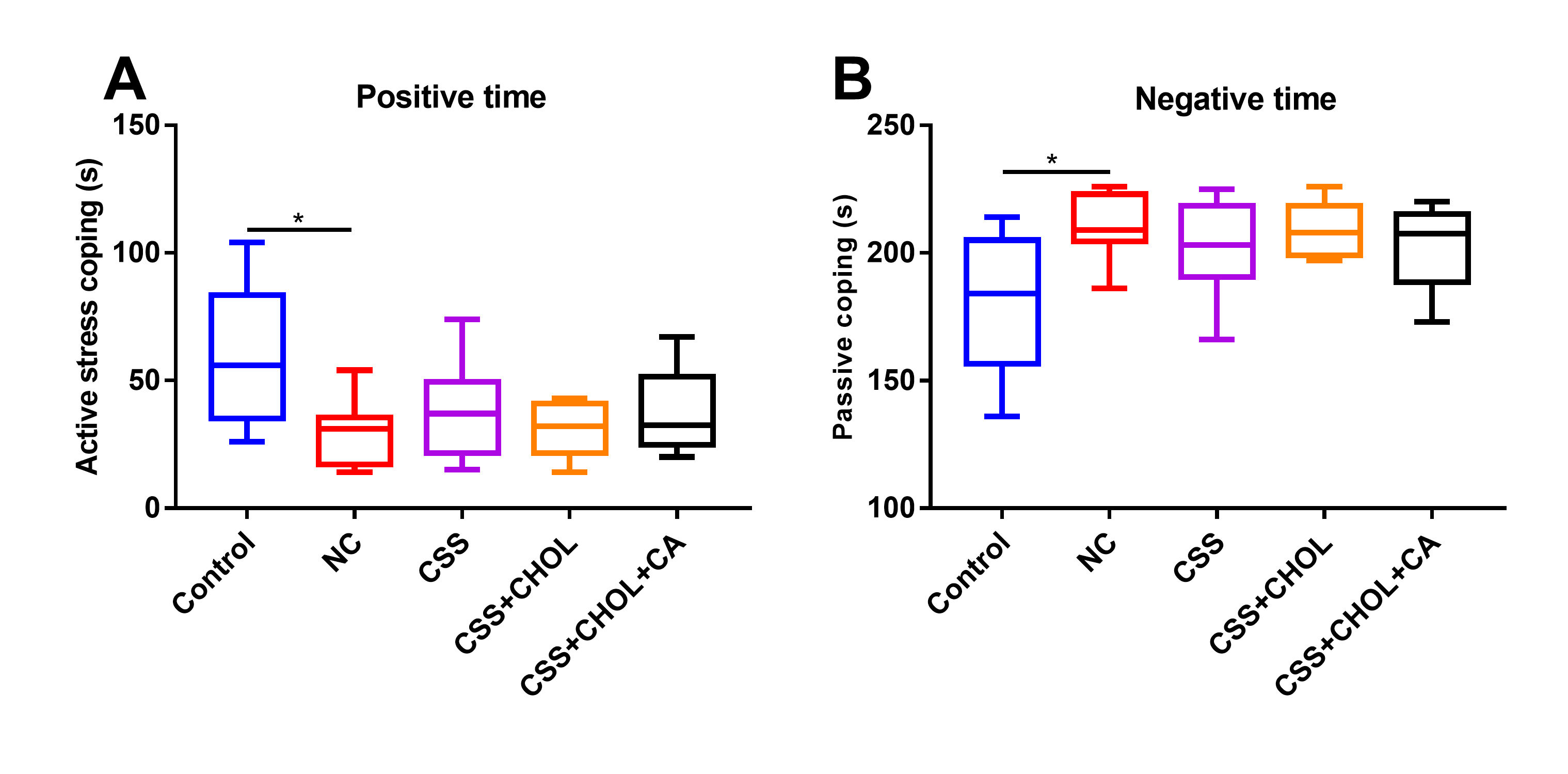


**Fig. S3. Spearman correlation analysis of serum bile acid and abundance of intestinal flora at genus level.** The heat map shows the correlation coefficient, with red indicating positive correlation and blue indicating negative correlation. An asterisk “*” denotes P<0.05.


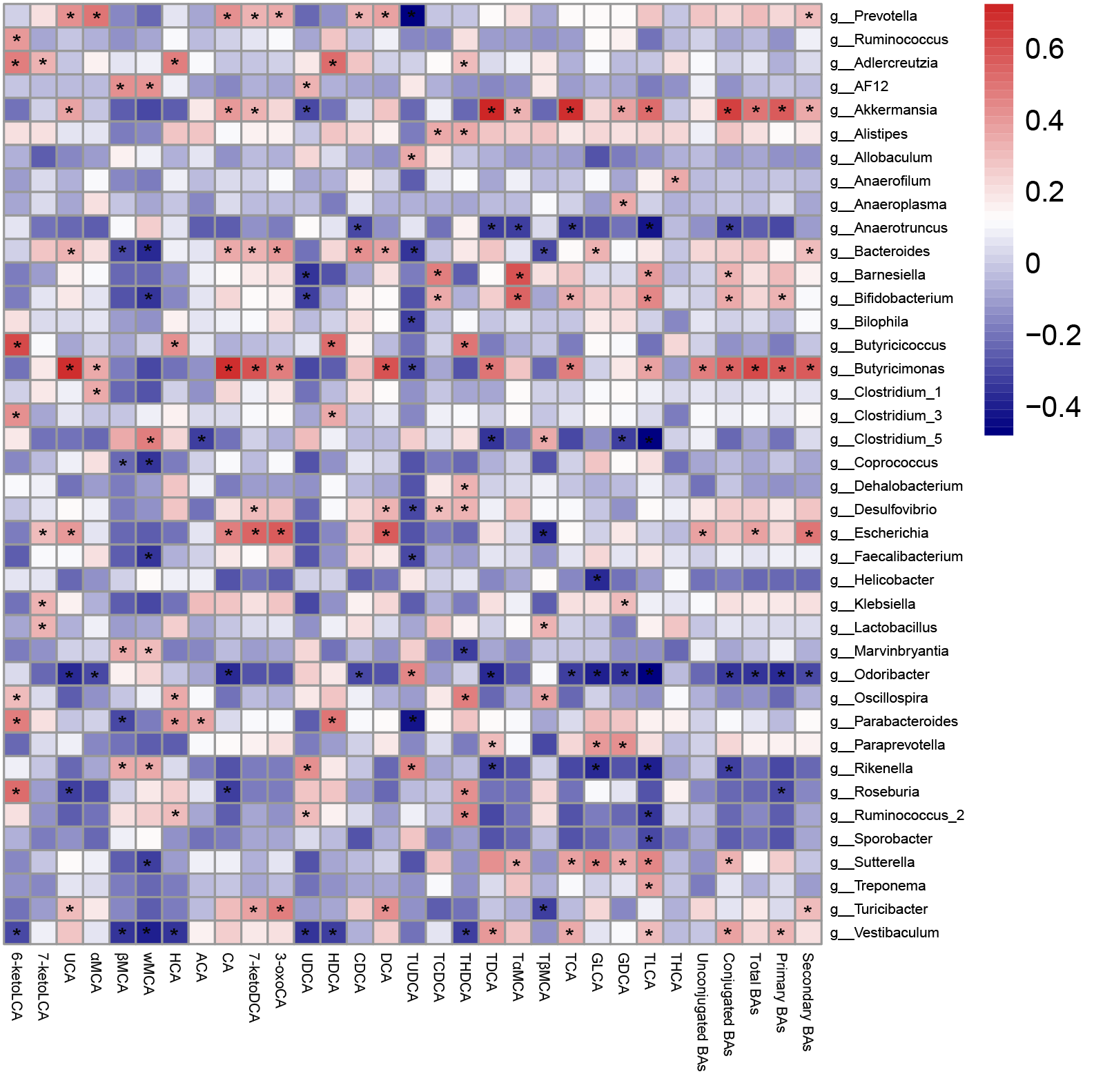


**Fig. S4 Effect of transplanting microbiota from CSS-treated mice into untreated mice on forced swim test outcomes in CUMS mice.** Mice were exposed to CUMS and treated with saline (NC group), CSS, or CSS along with the BA sequestrant CHOL. Control mice received saline but were not exposed to CUMS. Additional groups of CUMS mice were transplanted with fecal microbiota from CSS-treated groups (FMT) or filtered FMT (FFMT) to remove microbiota. Mice were placed in the forced swim test and the time spent struggling (A; positive time) or immobile (B; negative time) was recorded. All values are expressed as mean ± S.D. An asterisk “*” denotes P<0.05.


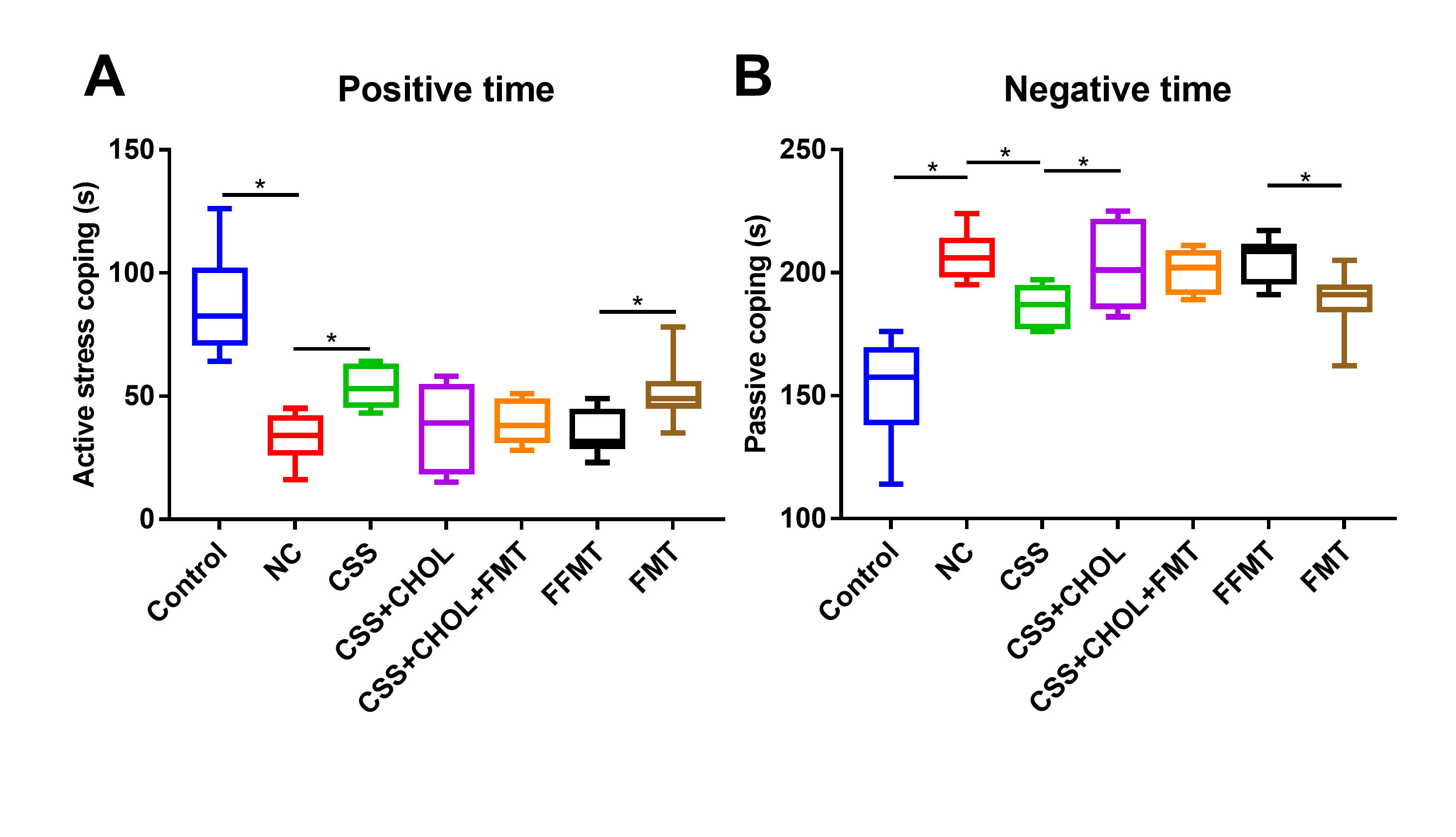


**Fig. S5. Correlation analysis comparing the concentration of serum 7-ketoDCA with the relative abundance of *Parabacteroides distasonis* in the colon.**


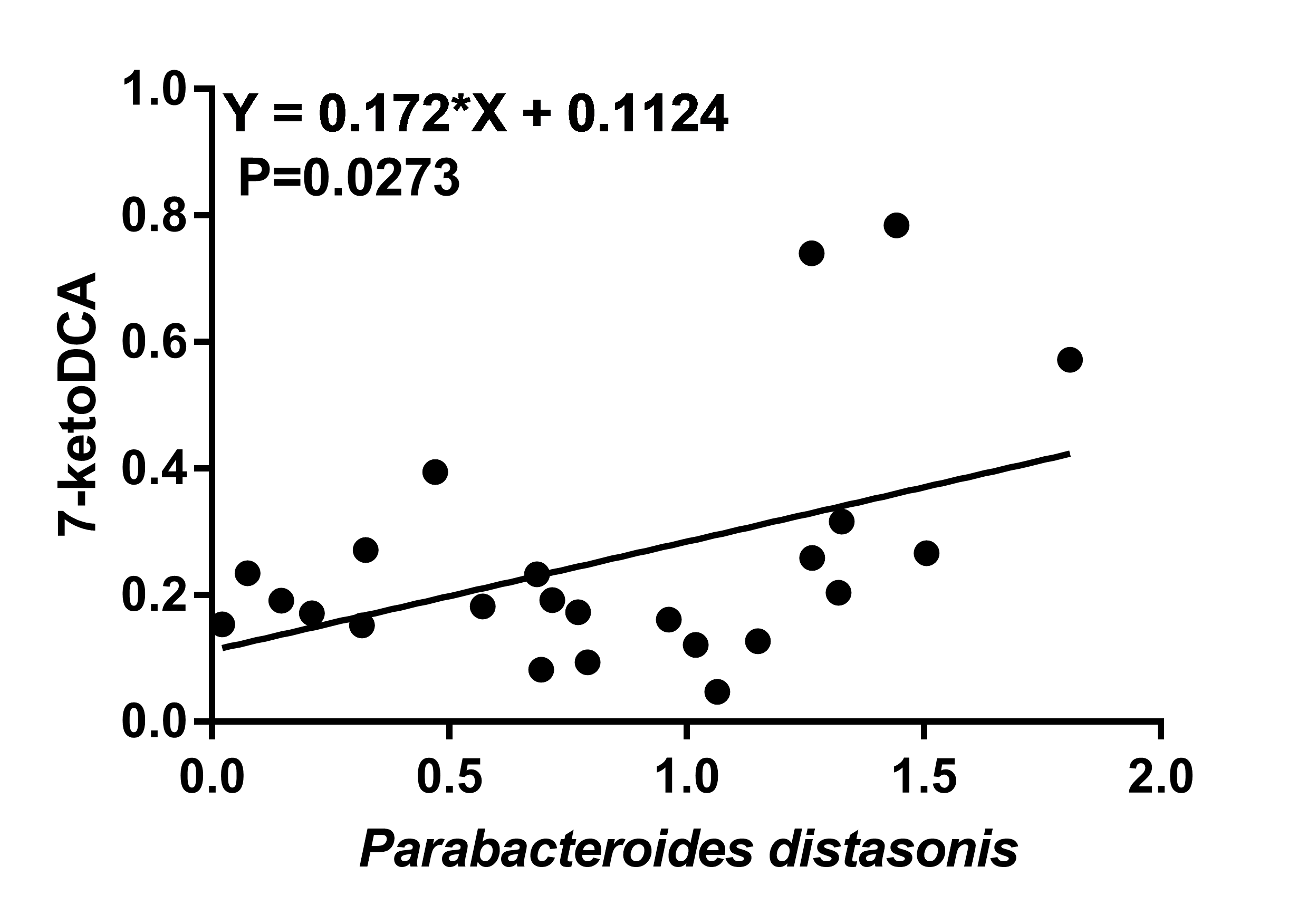


Table S1. Composition and corresponding ratio of Chaihu-Shugan-San.

| **Herb** | **Chinese name** | **Latin name** | **Ratio** | **Medicinal part** | **Batch number** |
| --- | --- | --- | --- | --- | --- |
| Bupleurum chinense DC. | Chai Hu | Radix Bupleuri | 4 | Root | 20051802 |
| Citrus reticulata Blanco | Chen Pi | Pericarpium Citri Reticulatae | 4 | Pericarp | 20071804 |
| Cyperus rotundus L. | Xiang Fu | Rhizoma Cyperi | 3 | Root | 20060202 |
| Citrus × aurantium L. | Zhi Qiao | Fructus Aurantii | 3 | Fruit | 20072111 |
| Ligusticum striatum DC. | Chuan Xiong | Rhizoma Chuanxiong | 3 | Rhizome | 20042708 |
| Paeonia lactiflora Pall. | Bai Shao | Radix Paeoniae Alba | 3 | Root | 20041711 |
| Glycyrrhiza uralensis Fisch. | Gan Cao | Radix Glycyrrhizae | 1 | Root and rhizome | 20040108 |

**Table S2. Bile acid liquid phase separation mobile phase gradient.**

| Time (min) | A (%) | B (%) | Flow (mL /min) |
| --- | --- | --- | --- |
| 0 | 95 | 5 | 0.3 |
| 1 | 92 | 8 | 0.3 |
| 10 | 86 | 14 | 0.3 |
| 11 | 75 | 25 | 0.3 |
| 15 | 75 | 25 | 0.3 |
| 16 | 50 | 50 | 0.3 |
| 19 | 50 | 50 | 0.3 |
| 22 | 5 | 95 | 0.3 |
| 24 | 5 | 95 | 0.3 |
| 24.5 | 95 | 5 | 0.3 |
| 26 | 95 | 5 | 0.3 |

**Table S3. Retention time and mass spectrometric parameters of bile acids.**

| Abbreviation | RT (min) Serum /Liver | Transition | DP/V | CE/V |
| --- | --- | --- | --- | --- |
| isoalloLCA | 18.76/18.94 | 375.3>375.3 | -200 | -30 |
| isoLCA | 21.11/20.84 | 375.3>375.3 | -200 | -30 |
| LCA | 20.64/21.36 | 375.3>375.3 | -200 | -30 |
| UDCA | 10.49/11.26 | 391.3>391.3 | -200 | -30 |
| HDCA | 11.52/12.15 | 391.3>391.3 | -200 | -30 |
| CDCA | 16.03/16.57 | 391.3>391.3 | -200 | -30 |
| DCA | 16.75/17 | 391.3>391.3 | -200 | -30 |
| GLCA | 17.36/17.44 | 432.3>74 | -147 | -79 |
| GUDCA | 7.68/8.31 | 448.3>74 | -180 | -75 |
| GHDCA | 8.07/8.79 | 448.3>74 | -180 | -75 |
| GCDCA | 13.24/13.54 | 448.3>74 | -180 | -75 |
| GDCA | 14.02/14.38 | 448.3>74 | -180 | -75 |
| GDHCA | 2.91/3.1 | 458.3>74 | -160 | -73 |
| GHCA | 6/6.54 | 464.3>74 | -150 | -80 |
| GCA | 8.99/9.73 | 464.3>74 | -150 | -80 |
| TLCA | 17.66/17.72 | 482.3>79.9 | -150 | -140 |
| TUDCA | 9.21/9.72 | 498.3>79.9 | -145 | -135 |
| THDCA | 9.27/10.56 | 498.3>79.9 | -145 | -135 |
| TCDCA | 14.09/14.46 | 498.3>79.9 | -145 | -135 |
| TDCA | 15.03/15.47 | 498.3>79.9 | -145 | -135 |
| TDHCA | 3.39/3.6 | 508.3>79.9 | -160 | -128 |
| TαMCA | 4.75/5.14 | 514.3>79.9 | -130 | -136 |
| TβMCA | 4.94/5.37 | 514.3>79.9 | -130 | -136 |
| THCA | 7.29/7.92 | 514.3>79.9 | -130 | -136 |
| TCA | 10.4/11.22 | 514.3>79.9 | -130 | -136 |
| DHLCA | 21.21/21.36 | 373.27>373.27 | -138 | -25 |
| UCA | 3.65/3.92 | 407.3>407.3 | -220 | -20 |
| ωMCA | 4.73/5.12 | 407.3>407.3 | -220 | -20 |
| αMCA | 5.19/5.6 | 407.3>407.3 | -220 | -20 |
| βMCA | 5.66/6.1 | 407.3>407.3 | -220 | -20 |
| HCA | 7.97/8.63 | 407.3>407.3 | -220 | -20 |
| ACA | 9.36/10.08 | 407.3>407.3 | -220 | -20 |
| CA | 10.49/11.29 | 407.3>407.3 | -220 | -20 |
| TLCA-3S | 12.05/12.35 | 280.8>97 | -115 | -32 |
| LCA-3S | 13.82/14.19 | 455.3>97 | -55 | -44 |
| GLCA-3S | 10.5/11.42 | 255.4>97 | -50 | -32 |
| 7-ketoDCA | 5.79/6.25 | 405.28>405.8 | -220 | -25 |
| 3-oxoCA | 8.69/9.31 | 405.28>405.8 | -220 | -25 |
| DHCA | 3.54/7.73 | 401.2>401.2 | -220 | -20 |
| 6-ketoLCA | 11.67/12.23 | 389.2>389.2 | -220 | -20 |
| 7-ketoLCA | 12.8/13.13 | 389.2>389.2 | -220 | -20 |
| apoCA | 13.4/13.76 | 389.2>389.2 | -128 | -28 |
| D4-CA | 10.51/11.3 | 411.2>411.2 | -150 | -14 |
| D4-LCA | 20.65/21.37 | 379.3>379.3 | -160 | -20 |
| D4-TCA | 10.4/11.21 | 518.2>80 | -137 | -143 |
| D4-GCA | 8.98/9.72 | 468.3>74.3 | -135 | -91 |

**Table S4. Bile acid working solution concentration.**

|  | Bile acid working solution concentration ng/mL |
| --- | --- |
| C1 | 25000/10000/5000/3000 |
| C2 | 15000/6000/3000/1800 |
| C3 | 7500/3000/1500/900 |
| C4 | 2500/1000/500/300 |
| C5 | 1250/500/250/150 |
| C6 | 500/200/100/60 |
| C7 | 125/50/25/15 |
| HQC | 20000/8000/4000/2400 |
| MQC | 5000/2000/1000/600 |
| LQC | 250/100/50/30 |
| LLOQ | 125/50/25/15 |
| IS | 1000 |

**Table S5. Retention time and mass spectrometric parameters of bile acids.**

| Bile acid | RT (min) Serum /Liver | Linear Range (ng/mL) | Linearity Serum /Liver | R^2^ Serum /Liver | LOD/LLOQ (ng/mL) | IS |
| --- | --- | --- | --- | --- | --- | --- |
| isoalloLCA | 18.76/18.94 | 2.5-500 | y=0.00883x+0.0083/y=0.00898x+0.0012 | 0.9936/0.9968 | 0.8/2.5 | D4-LCA |
| isoLCA | 21.11/20.84 | 2.5-500 | y=0.00135x-0.000147/y=0.0029x-0.01021 | 0.9926/0.989 | 0.8/2.5 | D4-LCA |
| LCA | 20.64/21.36 | 2.5-500 | y=0.0126x+0.027/y=0.00135x-0.00493 | 0.994/0.9409 | 0.8/2.5 | D4-LCA |
| UDCA | 10.49/11.26 | 2.5-500 | y=0.00777x-0.00318/y=0.0083x-0.00264 | 0.9994/0.995 | 0.8/2.5 | D4-CA |
| HDCA | 11.52/12.15 | 2.5-500 | y=0.0137x-0.00189/y=0.0045x+0.00838 | 0.9988/0.9962 | 0.8/2.5 | D4-CA |
| CDCA | 16.03/16.57 | 12.5-2500 | y=0.00823x+0.00131/y=0.0842x+0.00531 | 0.9988/0.9962 | 4/12.5 | D4-CA |
| DCA | 16.75/17 | 1.5-300 | y=0.0145x-0.00225/y=0.0141x+0.128 | 0.9946/0.992 | 0.5/1.5 | D4-CA |
| GLCA | 17.36/17.44 | 2.5-500 | y=0.0268x-0.00495/y=0.0253x+0.0021 | 0.995/0.996 | 0.8/2.5 | D4-GCA |
| GUDCA | 7.68/8.31 | 1.5-300 | y=0.0335x-0.00183/y=0.0352x+0.00276 | 0.9964/0.992 | 0.5/1.5 | D4-GCA |
| GHDCA | 8.07/8.79 | 2.5-500 | y=0.0129x-0.00371/y=0.0134x-0.0365 | 0.9974/0.995 | 0.8/2.5 | D4-GCA |
| GCDCA | 13.24/13.54 | 12.5-2500 | y=0.0153x-0.00892/y=0.0153x-0.0109 | 0.9992/0.9936 | 4/12.5 | D4-GCA |
| GDCA | 14.02/14.38 | 1.5-300 | y=0.0312x-0.00482/y=0.0335x-0.0588 | 0.9974/0.991 | 0.5/1.5 | D4-GCA |
| GDHCA | 2.91/3.1 | 2.5-500 | y=0.00631+0.000395/y=0.0066x-0.00177 | 0.9918/0.993 | 0.8/2.5 | D4-GCA |
| GHCA | 6/6.54 | 2.5-500 | y=0.0204x-0.00207/y=0.0202x-0.00387 | 0.9964/0.992 | 0.8/2.5 | D4-GCA |
| GCA | 8.99/9.73 | 12.5-2500 | y=0.016x+0.0455/y=0.0153x-0.00627 | 0.999/0.9944 | 4/12.5 | D4-GCA |
| TLCA | 17.66/17.72 | 2.5-500 | y=0.0457x-0.00301/y=0.0508x-0.00574 | 0.9946/0.992 | 0.8/2.5 | D4-TCA |
| TUDCA | 9.21/9.72 | 2.5-500 | y=0.0313x+0.248/y=0.0399x-0.0241 | 0.9992/0.9924 | 0.8/2.5 | D4-TCA |
| THDCA | 9.27/10.56 | 2.5-500 | y=0.0305x+0.217/y=0.0354x+0.0269 | 0.9986/0.9926 | 0.8/2.5 | D4-TCA |
| TCDCA | 14.09/14.46 | 2.5-500 | y=0.0287x+0.00724/y=0.00283x+0.00197 | 0.9916/0.9958 | 0.8/2.5 | D4-TCA |
| TDCA | 15.03/15.47 | 1.5-300 | y=0.0359x+0.0428/y=0.00342x+0.0303 | 0.9918/0.9974 | 0.5/1.5 | D4-TCA |
| TDHCA | 3.39/3.6 | 1.5-300 | y=0.00224x+6.37*10^5/y=0.00146x-0.0932 | 0.9958/0.9932 | 0.5/1.5 | D4-TCA |
| TαMCA | 4.75/5.14 | 12.5-2500 | y=0.0121x+0.00529/y=0.00113x+0.0024 | 0.9994/0.9934 | 4/12.5 | D4-TCA |
| TβMCA | 4.94/5.37 | 12.5-2500 | y=0.0247x+0.00289/y=0.00225x+0.0307 | 0.9966/0.9924 | 4/12.5 | D4-TCA |
| THCA | 7.29/7.92 | 1.5-300 | y=0.0224x-0.00756/y=0.00240x+0.000158 | 0.9972/0.9988 | 0.5/1.5 | D4-TCA |
| TCA | 10.4/11.22 | 12.5-2500 | y=0.0283x+0.027/y=0.00268x+0.0217 | 0.999/0.996 | 4/12.5 | D4-TCA |
| DHLCA | 21.21/21.36 | 5-1000 | y=0.0179x-0.00521/y=0.018x+0.00982 | 0.9958/0.9936 | 1.5/4.5 | D4-LCA |
| UCA | 3.65/3.92 | 1.5-300 | y=0.012x+0.0126/y=0.013x-0.00106 | 0.9952/0.9964 | 0.5/1.5 | D4-CA |
| ωMCA | 4.73/5.12 | 12.5-2500 | y=0.00823x+0.00297/y=0.00866x-0.0078 | 0.9952/0.9996 | 4/12.5 | D4-CA |
| αMCA | 5.19/5.6 | 12.5-2500 | y=0.0071x-2.82*10^5/y=0.00728x+0.00762 | 0.9978/0.9998 | 4/12.5 | D4-CA |
| βMCA | 5.66/6.1 | 12.5-2500 | y=0.0093x+0.0142/y=0.0091x+0.0371 | 0.9978/0.9996 | 4/12.5 | D4-CA |
| HCA | 7.97/8.63 | 1.5-300 | y=0.0112x-0.00129/y=0.0115x+0.00439 | 0.9966/0.9976 | 0.5/1.5 | D4-CA |
| ACA | 9.36/10.08 | 2.5-500 | y=0.00293x-0.072/y=0.0333x-0.0823 | 0.9936/0.9998 | 0.8/2.5 | D4-CA |
| CA | 10.49/11.29 | 12.5-2500 | y=0.0125x+0.0877/y=0.0132x+0.106 | 0.998/0.9994 | 4/12.5 | D4-CA |
| TLCA-3S | 12.05/12.35 | 5-1000 | y=0.0466x-0.022/y=0.00437x-0.00153 | 0.9918/0.9986 | 1.5/4.5 | D4-TCA |
| LCA-3S | 13.82/14.19 | 1.5-300 | y=0.0401x-0.00356/y=0.257x-0.000377 | 0.9926/0.9924 | 0.5/1.5 | D4-LCA |
| GLCA-3S | 10.5/11.42 | 5-1000 | y=0.0031x-0.00356/y=0.000467x-0.00113 | 0.9982/0.9938 | 1.5/4.5 | D4-GCA |
| 7-ketoDCA | 5.79/6.25 | 5-1000 | y=0.00752x+0.00302/y=0.00734x+0.00341 | 0.9988/0.9972 | 1.5/4.5 | D4-CA |
| 3-oxoCA | 8.69/9.31 | 5-1000 | y=0.00738x-0.00108/y=0.00726x+0.00319 | 0.999/0.999 | 1.5/4.5 | D4-CA |
| DHCA | 3.54/7.73 | 2.5-500 | y=0.00203x-0.00183/y=0.00199x-0.00133 | 0.9972/0.999 | 0.8/2.5 | D4-CA |
| 6-ketoLCA | 11.67/12.23 | 5-1000 | y=0.0151x+0.00958/y=0.0168x+0.00326 | 0.9986/0.9976 | 1.5/4.5 | D4-LCA |
| 7-ketoLCA | 12.8/13.13 | 5-1000 | y=0.0118x-0.00165/y=0.0116x+0.00106 | 0.9994/0.9986 | 1.5/4.5 | D4-LCA |
| apoCA | 13.4/13.76 | 5-1000 | y=0.0103x+0.0085/y=0.0106x+0.0118 | 0.994/0.996 | 1.5/4.5 | D4-CA |

**Table S6. Inter and intra-day precision and accuracy of 42 bile acid components in mouse serum.**

| Bile acid | LLOQ | | | | LQC | | | | MQC | | | | HQC | | | |
| --- | --- | --- | --- | --- | --- | --- | --- | --- | --- | --- | --- | --- | --- | --- | --- | --- |
|  | Intra-day (n=6) | | Inter-day (n=18) | | Intra-day (n=6) | | Inter-day (n=18) | | Intra-day (n=6) | | Inter-day (n=18) | | Intra-day (n=6) | | Inter-day (n=18) | |
|  | Accuracy | %RSD | Accuracy | %RSD | Accuracy | %RSD | Accuracy | %RSD | Accuracy | %RSD | Accuracy | %RSD | Accuracy | %RSD | Accuracy | %RSD |
| isoalloLCA | 87.1 | 3.58 | 98.4 | 6.24 | 97.08 | 13.33 | 96.29 | 1.15 | 95.65 | 5.26 | 95.95 | 0.44 | 98.25 | 4.89 | 98.58 | 0.48 |
| isoLCA | 97.5 | 9.11 | 102.13 | 6.4 | 95.72 | 13.97 | 95.13 | 0.87 | 97 | 14.43 | 99.78 | 3.93 | 108.93 | 9.45 | 106.8 | 2.82 |
| LCA | 96.64 | 5.59 | 94.8 | 2.74 | 93.14 | 4.07 | 98.94 | 8.29 | 103.2 | 6.03 | 102.43 | 1.07 | 101.3 | 2.99 | 101.61 | 0.43 |
| UDCA | 106.8 | 8.76 | 106.75 | 0.07 | 100.85 | 9.8 | 96.86 | 5.82 | 99.95 | 3.84 | 100.15 | 0.28 | 103.55 | 2.92 | 104.44 | 1.21 |
| HDCA | 87.82 | 9.65 | 89.23 | 2.23 | 95.07 | 9.06 | 96.56 | 2.18 | 93.68 | 5.57 | 95.16 | 2.2 | 90.88 | 2.23 | 91.51 | 0.97 |
| CDCA | 95.5 | 6.78 | 98 | 3.61 | 101.5 | 5.65 | 101.65 | 0.21 | 98.3 | 3.19 | 98.45 | 0.22 | 113.3 | 2.73 | 103.57 | 0.33 |
| DCA | 102 | 8.16 | 104.07 | 2.81 | 109.96 | 12.32 | 110.71 | 0.97 | 99.04 | 4.99 | 100.8 | 2.47 | 95.6 | 3.39 | 96.47 | 1.27 |
| GLCA | 101.44 | 3.4 | 100.28 | 1.64 | 113.55 | 12.74 | 104.25 | 0.87 | 95.8 | 5.34 | 99.78 | 5.64 | 100.71 | 12.51 | 103.5 | 3.82 |
| GUDCA | 103.18 | 5.42 | 99.76 | 4.86 | 98.24 | 14.93 | 100.24 | 2.82 | 98.16 | 6.56 | 102 | 5.32 | 94.8 | 7.32 | 97.67 | 4.15 |
| GHDCA | 103 | 6.96 | 108.95 | 7.72 | 91.16 | 6.61 | 90.74 | 0.65 | 100.84 | 8.03 | 104.36 | 4.76 | 99.78 | 5.84 | 102.56 | 3.83 |
| GCDCA | 102.46 | 6.02 | 102.66 | 0.28 | 97.25 | 6.77 | 101.55 | 5.99 | 100.7 | 6.09 | 104.35 | 4.95 | 95.36 | 6.47 | 98.95 | 5.13 |
| GDCA | 94.6 | 3.86 | 95.4 | 1.19 | 104.24 | 9.94 | 108.46 | 5.5 | 100.72 | 3.82 | 104.72 | 5.4 | 101.92 | 6.96 | 103.76 | 2.51 |
| GDHCA | 101.68 | 6.19 | 100.08 | 2.26 | 95.18 | 2.08 | 97.86 | 3.87 | 88.28 | 9.24 | 94.08 | 8.72 | 95.2 | 11.59 | 98.9 | 5.29 |
| GHCA | 105.2 | 1.67 | 100.83 | 6.12 | 90.56 | 9.42 | 95.86 | 7.82 | 92.4 | 5.95 | 97.72 | 7.7 | 94.13 | 9.71 | 96.55 | 3.54 |
| GCA | 102.44 | 7.27 | 107.78 | 7.01 | 103.4 | 1.33 | 106.65 | 4.31 | 103.8 | 5.04 | 108.95 | 6.68 | 106.8 | 10.29 | 109.07 | 2.94 |
| TLCA | 99.3 | 6.72 | 101.55 | 3.13 | 90.4 | 9.69 | 98.36 | 11.44 | 94.32 | 6.67 | 102.42 | 11.18 | 100.04 | 6.36 | 104.09 | 5.5 |
| TUDCA | 108.2 | 4.81 | 107.35 | 1.12 | 95.42 | 7.8 | 89.18 | 14.72 | 94.27 | 4.63 | 99.16 | 6.97 | 95.96 | 7.67 | 98.13 | 3.13 |
| THDCA | 98.13 | 1.71 | 99.03 | 1.29 | 96.74 | 13.09 | 100.07 | 4.71 | 98 | 2.69 | 103.35 | 7.32 | 98.35 | 7.05 | 101.38 | 4.23 |
| TCDCA | 102.56 | 2.32 | 101.16 | 1.96 | 87.9 | 12.52 | 92.76 | 7.41 | 97.2 | 6.83 | 103.7 | 8.86 | 97.6 | 7.65 | 100.73 | 4.39 |
| TDCA | 90.88 | 3.49 | 92.29 | 2.16 | 104.88 | 9.07 | 108.26 | 4.42 | 102 | 4.67 | 104.52 | 3.41 | 100.32 | 7.53 | 103.36 | 4.16 |
| TDHCA | 97.97 | 4.67 | 96.53 | 2.1 | 86.88 | 5.13 | 88.08 | 1.93 | 89.68 | 9.64 | 93.92 | 6.38 | 91.84 | 9.45 | 94.12 | 3.43 |
| TαMCA | 102.72 | 3.32 | 102.35 | 0.51 | 89.1 | 7.59 | 87.2 | 8.27 | 89.4 | 6.16 | 87.65 | 6.86 | 93.76 | 6.76 | 95.95 | 3.22 |
| TβMCA | 93.56 | 6.86 | 100.96 | 10.37 | 87.75 | 4.23 | 94.68 | 10.34 | 94.5 | 3.53 | 98.95 | 6.36 | 98.72 | 5.92 | 100.69 | 2.77 |
| THCA | 102.62 | 9.85 | 97.87 | 6.86 | 114.07 | 4.51 | 106.04 | 10.72 | 89.73 | 11.43 | 97.23 | 10.91 | 99.12 | 8.31 | 102.49 | 4.65 |
| TCA | 100.17 | 10.68 | 103.67 | 4.77 | 98.3 | 6.1 | 98.1 | 0.29 | 94.8 | 5.28 | 97.65 | 4.13 | 93.68 | 7.72 | 95.17 | 2.22 |
| DHLCA | 101.65 | 5.28 | 102.76 | 1.53 | 94.27 | 7.82 | 97.47 | 4.64 | 95.7 | 4.48 | 94.68 | 1.53 | 101.89 | 5.14 | 102.92 | 1.42 |
| UCA | 92.76 | 12.13 | 96.29 | 5.18 | 88 | 8.66 | 92.16 | 6.38 | 97.36 | 2.87 | 97.56 | 0.29 | 98.08 | 4.06 | 98.97 | 1.28 |
| ωMCA | 101.6 | 1.6 | 109.8 | 2.12 | 99.5 | 1.55 | 100.73 | 1.72 | 97.5 | 1.92 | 98.6 | 1.58 | 107.9 | 4.6 | 109.12 | 1.58 |
| αMCA | 108.8 | 6.11 | 117 | 2.18 | 104.05 | 4.12 | 104.9 | 1.15 | 104.1 | 2.24 | 104.75 | 0.88 | 106.9 | 3.81 | 107.45 | 0.72 |
| βMCA | 96.18 | 2.55 | 95.14 | 1.55 | 90.75 | 4.19 | 90.75 | 0 | 98.6 | 2.47 | 100.05 | 2.05 | 105.5 | 2.44 | 106.17 | 0.89 |
| HCA | 92.66 | 4.95 | 90.17 | 3.91 | 88.92 | 7.32 | 90.68 | 2.74 | 96.48 | 2.18 | 98.24 | 2.53 | 94.8 | 3 | 95.6 | 1.18 |
| ACA | 107.6 | 0.34 | 108.87 | 1.65 | 98 | 2.96 | 99.88 | 2.67 | 104.65 | 2.28 | 101.63 | 4.21 | 100.6 | 7.81 | 103.83 | 4.4 |
| CA | 95.8 | 4.33 | 98.65 | 4.09 | 92.35 | 9.8 | 89.08 | 5.2 | 99.2 | 1.66 | 100.55 | 1.9 | 107.1 | 4.3 | 107.8 | 0.92 |
| TLCA-3S | 100.76 | 10.61 | 102.34 | 2.18 | 99.6 | 7.55 | 100.5 | 1.27 | 100.24 | 7.78 | 104.02 | 5.14 | 96.18 | 12.58 | 100.03 | 5.44 |
| LCA-3S | 113 | 6.97 | 106 | 3.66 | 96.4 | 11.14 | 106.34 | 3.22 | 102.48 | 1.55 | 108.08 | 7.33 | 90.4 | 8.39 | 94.64 | 6.34 |
| GLCA-3S | 102.08 | 3.75 | 99.6 | 3.52 | 110.63 | 9.65 | 109.76 | 4.65 | 103.48 | 13.42 | 101.65 | 2.55 | 92.18 | 14.69 | 91.8 | 0.58 |
| 7-ketoDCA | 98.9 | 5.35 | 99.93 | 1.45 | 103.2 | 3.43 | 105.87 | 3.56 | 101.53 | 2.21 | 101.83 | 0.42 | 92.53 | 4.28 | 93.45 | 1.4 |
| 3-oxoCA | 101.8 | 3.66 | 101.32 | 0.67 | 111.33 | 4.02 | 113.2 | 2.33 | 102.35 | 4.36 | 102.15 | 0.28 | 98.63 | 5.91 | 99.44 | 1.16 |
| DHCA | 95.89 | 7.7 | 97.64 | 2.53 | 91.9 | 11.65 | 88.44 | 5.54 | 97.71 | 4.28 | 101.74 | 5.6 | 101.65 | 4.67 | 100.7 | 1.33 |
| 6-ketoLCA | 98.24 | 4.74 | 98.94 | 1 | 103.87 | 4.09 | 102.93 | 1.28 | 94.63 | 5.99 | 95.14 | 0.76 | 94.73 | 2.58 | 95.76 | 1.53 |
| 7-ketoLCA | 101.07 | 3.2 | 100.13 | 1.33 | 105.07 | 5.95 | 105.8 | 0.98 | 97.83 | 6.23 | 98.33 | 0.72 | 97.38 | 1.35 | 97.44 | 0.09 |
| apoCA | 104.63 | 2.28 | 103.43 | 1.64 | 102.4 | 13.87 | 103.07 | 0.91 | 96.9 | 3.58 | 98 | 1.59 | 99.85 | 4.11 | 101.18 | 1.85 |

**Table S7. Inter and intra-day precision and accuracy of 42 bile acid components in mouse liver.**

| Bile acid | LLOQ | | | | LQC | | | | MQC | | | | HQC | | | |
| --- | --- | --- | --- | --- | --- | --- | --- | --- | --- | --- | --- | --- | --- | --- | --- | --- |
|  | Intra-day (n=6) | | Inter-day (n=18) | | Intra-day (n=6) | | Inter-day (n=18) | | Intra-day (n=6) | | Inter-day (n=18) | | Intra-day (n=6) | | Inter-day (n=18) | |
|  | Accuracy | %RSD | Accuracy | %RSD | Accuracy | %RSD | Accuracy | %RSD | Accuracy | %RSD | Accuracy | %RSD | Accuracy | %RSD | Accuracy | %RSD |
| isoalloLCA | 111.04 | 6.58 | 113.94 | 3.6 | 95.25 | 7.66 | 96.86 | 2.35 | 88.48 | 5.25 | 91.64 | 4.88 | 100.4 | 6.92 | 100.62 | 0.3 |
| isoLCA | 96.88 | 5.29 | 100 | 4.42 | 101.5 | 3.18 | 109.42 | 14.31 | 102.83 | 4.04 | 104.75 | 2.59 | 107 | 12.09 | 107.94 | 0.97 |
| LCA | 109.53 | 9.13 | 104.73 | 6.48 | 96.84 | 5.77 | 97.98 | 1.64 | 91.82 | 5.61 | 93.21 | 2.11 | 100.8 | 7.92 | 99.53 | 1.81 |
| UDCA | 101.7 | 3.69 | 102.16 | 0.64 | 94.68 | 4.04 | 93.19 | 2.27 | 99.78 | 5.71 | 100.18 | 0.56 | 102.5 | 6.36 | 102.92 | 0.57 |
| HDCA | 109.1 | 4.09 | 107.55 | 2.04 | 102.1 | 4.94 | 101.85 | 0.35 | 113.22 | 4.69 | 114.56 | 4.04 | 89.28 | 5.19 | 90.31 | 1.61 |
| CDCA | 108.67 | 3.5 | 109.83 | 1.5 | 101.13 | 5.66 | 99.88 | 1.77 | 96.69 | 4.01 | 98.06 | 1.98 | 101.6 | 5.28 | 101.97 | 0.51 |
| DCA | 98.5 | 3.95 | 98.79 | 0.41 | 96.9 | 8.11 | 96.6 | 0.44 | 103.22 | 10.23 | 106.86 | 7.7 | 89.28 | 5.45 | 89.51 | 0.36 |
| GLCA | 106.54 | 5.13 | 102.76 | 5.2 | 95.44 | 6.43 | 98.03 | 3.73 | 86.12 | 8.41 | 89.74 | 5.7 | 92.44 | 6.33 | 93.96 | 2.29 |
| GUDCA | 95.21 | 4.54 | 99.69 | 6.35 | 97.1 | 3.27 | 97.4 | 0.44 | 101.25 | 10.13 | 93.8 | 11.23 | 88.48 | 6.61 | 89.37 | 1.41 |
| GHDCA | 95.8 | 8.47 | 100.95 | 7.21 | 100.11 | 4.73 | 100.69 | 0.82 | 87.53 | 14.99 | 87.43 | 0.18 | 87.78 | 4.75 | 88.67 | 1.42 |
| GCDCA | 114.86 | 10.06 | 100.53 | 6.65 | 103.5 | 6.3 | 103.81 | 0.43 | 96.38 | 2.81 | 99.34 | 4.23 | 89.44 | 5.95 | 88.59 | 0.25 |
| GDCA | 104.5 | 7.47 | 101.5 | 4.19 | 97.7 | 7.05 | 100.1 | 3.39 | 102.3 | 8.21 | 106.53 | 5.61 | 89.52 | 6.87 | 88.56 | 0.07 |
| GDHCA | 99.14 | 1.49 | 100.37 | 1.73 | 97.39 | 9.28 | 100.11 | 3.85 | 86.73 | 10.72 | 85.63 | 1.82 | 88.52 | 2.26 | 89.23 | 1.12 |
| GHCA | 101.93 | 5.41 | 100.07 | 2.64 | 93.61 | 5.94 | 93 | 0.93 | 85.35 | 3.08 | 88.31 | 5.35 | 87.42 | 5.32 | 88.08 | 1.06 |
| GCA | 125.6 | 6.91 | 127 | 1.56 | 105.38 | 6.06 | 104.63 | 1.01 | 94.13 | 3.15 | 97.84 | 5.38 | 98.4 | 5.3 | 99.12 | 1.02 |
| TLCA | 106.5 | 8.95 | 102.4 | 5.66 | 91.56 | 3.04 | 94 | 3.68 | 89.75 | 10.71 | 84.66 | 8.21 | 86.36 | 8.56 | 88.28 | 3.08 |
| TUDCA | 109.9 | 4.34 | 109.25 | 0.84 | 96.11 | 5.34 | 96.17 | 0.08 | 89.78 | 10.35 | 85.83 | 8.32 | 88.89 | 2.59 | 89.48 | 0.94 |
| THDCA | 98.47 | 4.23 | 99.87 | 1.98 | 101.44 | 7.69 | 102.25 | 1.11 | 107.35 | 14.15 | 109.22 | 2.41 | 99.2 | 6.34 | 99.77 | 0.8 |
| TCDCA | 101.44 | 4.18 | 101.15 | 0.41 | 99.39 | 8.05 | 98.08 | 1.88 | 94.58 | 7.41 | 92.29 | 3.51 | 88.76 | 5.89 | 90.23 | 2.31 |
| TDCA | 105.63 | 4.19 | 103.4 | 3.05 | 107.3 | 11.54 | 104.55 | 3.72 | 105.8 | 9.01 | 103.33 | 3.39 | 88.08 | 6.55 | 88.51 | 0.68 |
| TDHCA | 100 | 12.88 | 101.13 | 1.58 | 112 | 7.62 | 109.2 | 3.63 | 94.95 | 4.47 | 94.43 | 0.79 | 97.36 | 4.47 | 99.28 | 2.73 |
| TαMCA | 111 | 8.31 | 112.1 | 1.39 | 102.38 | 7.89 | 101.75 | 0.87 | 90.38 | 6.74 | 94.66 | 6.4 | 85.04 | 4.71 | 87.25 | 3.59 |
| TβMCA | 99 | 9.25 | 97.3 | 2.47 | 90.25 | 5.68 | 90.06 | 0.29 | 89.56 | 7.48 | 85.28 | 2.85 | 88.64 | 1.88 | 88.25 | 0.62 |
| THCA | 103.42 | 6.65 | 103.4 | 0.03 | 108.8 | 7.54 | 107.85 | 1.25 | 108.95 | 11.52 | 103 | 8.17 | 87.2 | 5.48 | 88 | 1.29 |
| TCA | 89.13 | 4.03 | 86.16 | 8.25 | 101.63 | 4.59 | 100.44 | 1.67 | 92.44 | 6.49 | 96.16 | 5.47 | 89.68 | 6.13 | 86.31 | 4.3 |
| DHLCA | 102.75 | 3.79 | 99.18 | 5.1 | 97.71 | 10.16 | 96.25 | 2.14 | 105.5 | 11.63 | 104.83 | 0.9 | 113.94 | 7.69 | 112.8 | 1.43 |
| UCA | 110.06 | 10.65 | 111.54 | 1.88 | 95 | 6.56 | 95.1 | 0.15 | 93.4 | 5.81 | 95.1 | 2.53 | 94.64 | 3.72 | 95.79 | 1.69 |
| ωMCA | 110.2 | 4.51 | 110 | 0.26 | 99.75 | 4.75 | 99.63 | 0.18 | 101.19 | 3.67 | 99.34 | 2.62 | 103.4 | 2.4 | 104.37 | 1.31 |
| αMCA | 106.68 | 7.43 | 108.04 | 1.63 | 104.63 | 7.22 | 104.56 | 0.08 | 101.19 | 4.87 | 104.03 | 3.87 | 102.7 | 3.7 | 103.43 | 1 |
| βMCA | 112.98 | 13.71 | 110.25 | 3.5 | 101.25 | 5.91 | 103 | 2.4 | 105.94 | 5.94 | 103.88 | 2.81 | 100.2 | 2.61 | 100.68 | 0.68 |
| HCA | 93.47 | 3.95 | 98 | 6.54 | 100.3 | 7.05 | 101.1 | 1.12 | 106.05 | 6.72 | 103.18 | 3.94 | 89.44 | 4.25 | 89.92 | 0.75 |
| ACA | 100.9 | 6.07 | 106.25 | 7.12 | 95.06 | 3.38 | 98.53 | 4.98 | 98.14 | 13.94 | 99.38 | 1.75 | 91.52 | 5.31 | 92.73 | 1.84 |
| CA | 106.8 | 12.37 | 106.38 | 0.51 | 102.75 | 5.65 | 102.56 | 0.26 | 106.56 | 4.24 | 105.41 | 1.55 | 102.1 | 4.8 | 103.05 | 1.3 |
| TLCA-3S | 104.84 | 11.3 | 102.1 | 3.8 | 89.03 | 8.88 | 91.35 | 3.59 | 809.38 | 10.24 | 89.81 | 4.16 | 93.48 | 6.37 | 94.74 | 1.88 |
| LCA-3S | 97.87 | 7.88 | 97.27 | 0.87 | 94.4 | 5.96 | 94.3 | 0.15 | 89.95 | 13.78 | 93.45 | 5.3 | 89.2 | 4.46 | 89.93 | 1.3 |
| GLCA-3S | 106.22 | 9.63 | 103.43 | 3.81 | 98 | 11.04 | 99.36 | 1.94 | 108.88 | 3.56 | 100.13 | 12.36 | 90.05 | 3.34 | 89.67 | 0.6 |
| 7-ketoDCA | 101.54 | 2.73 | 99.51 | 2.87 | 94.79 | 4.12 | 97.72 | 4.24 | 104.5 | 7.64 | 105.58 | 1.45 | 90.33 | 5.13 | 90.45 | 0.2 |
| 3-oxoCA | 97.28 | 4.77 | 98.5 | 1.75 | 93.68 | 3.87 | 95.01 | 1.99 | 107.5 | 13.66 | 108.42 | 1.2 | 94.63 | 4.84 | 95.42 | 1.17 |
| DHCA | 100.34 | 4 | 96.57 | 5.52 | 102.69 | 5.43 | 99.72 | 4.21 | 86.72 | 8.34 | 87.27 | 0.89 | 106.25 | 4.54 | 106.88 | 0.83 |
| 6-ketoLCA | 108.32 | 7.68 | 112.84 | 5.66 | 100.66 | 3.46 | 100.34 | 0.44 | 109.17 | 5.36 | 112.75 | 4.49 | 90.5 | 6.7 | 91.04 | 0.84 |
| 7-ketoLCA | 101.72 | 7.57 | 106.5 | 6.34 | 103.41 | 5.34 | 103.53 | 0.17 | 108.5 | 5.62 | 110.33 | 2.35 | 94.71 | 1.51 | 94.63 | 0.13 |
| apoCA | 97.91 | 1.25 | 98.98 | 1.53 | 102.69 | 5.64 | 100.89 | 2.52 | 94.33 | 7.9 | 96.33 | 2.94 | 96.85 | 1.94 | 97.43 | 0.83 |

**Table S8. Matrix effect of 42 bile acid components in mouse serum and liver.**

| Bile acid | Serum Matrix Effects | | | | | | Liver Matrix Effects | | | | | |
| --- | --- | --- | --- | --- | --- | --- | --- | --- | --- | --- | --- | --- |
|  | LC (n = 6) | | MC (n = 6) | | HC (n = 6) | | LC (n = 6) | | MC (n = 6) | | HC (n = 6) | |
| isoalloLCA | 101.13±9.14 | 9.03 | 100.14±9.61 | 4.44 | 105.81±4.69 | 4.44 | 98.38±6.55 | 6.66 | 94.54±12.07 | 12.77 | 102.18±4.35 | 4.25 |
| isoLCA | 103.31±2.72 | 2.63 | 105.75±3.95 | 3.5 | 107.32±3.75 | 3.5 | 102.9±6.1 | 5.93 | 99.19±5.15 | 5.19 | 108.38±6.73 | 6.21 |
| LCA | 99.24±8.25 | 8.31 | 104.19±4.96 | 3.48 | 108.89±3.79 | 3.48 | 101.62±9.03 | 8.89 | 99.73±9.18 | 9.2 | 107.93±2.48 | 2.3 |
| UDCA | 95.71±9.29 | 9.71 | 103.63±1.5 | 1.18 | 105.91±1.25 | 1.18 | 99.6±7.92 | 7.96 | 99.62±9.04 | 9.07 | 105.02±2.76 | 2.63 |
| HDCA | 99.16±11.46 | 11.56 | 98.62±6.96 | 0.88 | 103.06±0.91 | 0.88 | 109.12±5.07 | 4.65 | 99.62±12.29 | 12.33 | 92.21±7.04 | 7.63 |
| CDCA | 99.95±11.42 | 11.43 | 102.82±6.74 | 3.07 | 109.46±3.36 | 3.07 | 97.69±8.54 | 8.75 | 102.29±11.01 | 10.77 | 107.82±0.91 | 0.84 |
| DCA | 95.46±6.03 | 6.31 | 99.63±7.53 | 1.02 | 107.69±1.1 | 1.02 | 99.84±12.4 | 12.42 | 100.68±6.53 | 6.49 | 107.1±6.67 | 6.23 |
| GLCA | 100.26±6.97 | 6.95 | 110.58±3.73 | 5.4 | 108.46±5.86 | 5.4 | 103.99±5.1 | 4.91 | 107.11±4.68 | 4.37 | 105.99±5.22 | 4.92 |
| GUDCA | 100.09±6.87 | 6.81 | 110.85±2.61 | 6.28 | 105.82±6.65 | 6.28 | 101.7±5.8 | 5.7 | 109.64±3.7 | 3.37 | 104.31±2.67 | 2.56 |
| GHDCA | 100.47±10.01 | 9.96 | 103.04±8.68 | 1.93 | 108.34±2.09 | 1.93 | 98.53±8.14 | 8.26 | 102.07±9.43 | 9.24 | 102.98±2.43 | 2.36 |
| GCDCA | 100.61±11.18 | 11.11 | 109.48±4.19 | 5.08 | 108.4±5.51 | 5.08 | 102.81±1.85 | 1.8 | 107.81±5.42 | 5.03 | 95.77±7.22 | 7.54 |
| GDCA | 100.44±13.92 | 13.86 | 105.88±6.04 | 2.17 | 107.5±2.33 | 2.17 | 105.2±3.97 | 3.77 | 99.98±3.92 | 3.92 | 104.05±1.73 | 1.67 |
| GDHCA | 103.8±5.24 | 5.04 | 102.78±12.85 | 2.6 | 102.07±2.66 | 2.6 | 95.63±9.83 | 10.27 | 99.77±10.68 | 10.71 | 106.83±4.79 | 4.48 |
| GHCA | 95.91±9.59 | 10 | 105±3.68 | 3.95 | 106.86±4.23 | 3.95 | 103.25±4.77 | 4.62 | 103.43±2.49 | 2.41 | 103.8±0.56 | 0.54 |
| GCA | 101.11±10.7 | 10.58 | 107.08±6.74 | 1.67 | 106.02±1.77 | 1.67 | 105.78±3.59 | 3.4 | 100.25±8.84 | 8.82 | 107.85±3.86 | 3.58 |
| TLCA | 95.98±7.14 | 7.44 | 98.65±8.78 | 2.2 | 110.83±2.44 | 2.2 | 104.61±5.86 | 5.6 | 109.26±2.04 | 1.87 | 103.13±6.84 | 6.63 |
| TUDCA | 97.18±9.36 | 9.63 | 98.55±8.15 | 6.02 | 101.28±6.1 | 6.02 | 98.71±7.61 | 7.71 | 103.9±4.98 | 4.8 | 97.36±8.5 | 8.73 |
| THDCA | 96.07±6.05 | 6.29 | 104.27±7.26 | 8.58 | 100.16±8.59 | 8.58 | 99.82±3.51 | 3.52 | 106.59±4.39 | 4.12 | 99.5±9.16 | 9.21 |
| TCDCA | 97.36±6.86 | 7.05 | 102.65±2.8 | 1.02 | 102.06±1.04 | 1.02 | 101.75±4.65 | 4.57 | 102.71±11.05 | 10.76 | 103.85±6.26 | 6.03 |
| TDCA | 92.03±2.4 | 2.61 | 106.39±7.44 | 5.08 | 100.71±5.11 | 5.08 | 95.85±9.94 | 10.37 | 105.62±3.37 | 3.19 | 100.69±6.11 | 6.07 |
| TDHCA | 89.21±2.24 | 2.51 | 94.71±10.07 | 13.09 | 94.27±12.34 | 13.09 | 94.09±7.89 | 8.39 | 91.1±4.43 | 4.86 | 94.51±11.39 | 12.05 |
| TαMCA | 97±5.55 | 5.72 | 105.92±6.06 | 2.31 | 100.69±2.33 | 2.31 | 99.65±12.5 | 12.54 | 103.15±9.83 | 9.52 | 94.42±9.55 | 10.12 |
| TβMCA | 97.54±8.77 | 8.99 | 99.94±2.67 | 2.94 | 101.11±2.97 | 2.94 | 101.54±13.79 | 13.58 | 106.53±8.05 | 7.55 | 105.28±8.19 | 7.78 |
| THCA | 88.31±1.81 | 2.05 | 97.73±8.51 | 4.88 | 98.03±4.79 | 4.88 | 99.88±12.33 | 12.34 | 100.2±8.39 | 8.38 | 95.16±7.72 | 8.12 |
| TCA | 98.47±10.99 | 11.16 | 99.69±2.52 | 3.38 | 103.78±3.51 | 3.38 | 109.35±2.05 | 1.88 | 105.8±4.45 | 4.2 | 101.88±6.51 | 6.39 |
| DHLCA | 97.81±3.66 | 3.74 | 104.35±7.22 | 2.65 | 104.74±2.78 | 2.65 | 103.06±5.21 | 5.06 | 95.08±9.99 | 10.51 | 109.02±4.47 | 4.1 |
| UCA | 102.14±13.14 | 12.87 | 99.32±4.08 | 6.28 | 98.04±6.16 | 6.28 | 99.75±9.83 | 9.85 | 99.38±9.96 | 10.02 | 101.5±10.81 | 10.65 |
| ωMCA | 93.74±7.37 | 7.86 | 98.52±1.05 | 1.22 | 101.55±1.24 | 1.22 | 102.3±10.23 | 10 | 102.28±10.5 | 10.26 | 103.96±8.62 | 8.29 |
| αMCA | 97.6±7.62 | 7.81 | 98.17±3.36 | 2.4 | 100.33±2.41 | 2.4 | 97.49±10.68 | 10.95 | 99.86±9.2 | 9.21 | 99.3±6.31 | 6.36 |
| βMCA | 91.62±6.8 | 7.42 | 98.34±4.08 | 2.53 | 105.64±2.67 | 2.53 | 103.3±13.98 | 13.54 | 1032±12.39 | 12 | 107.1±6.9 | 6.45 |
| HCA | 97.03±9.73 | 10.03 | 99.29±5.28 | 2.48 | 101.59±2.52 | 2.48 | 104.66±15.73 | 15.03 | 102.8±11.47 | 11.16 | 104.07±6.44 | 6.19 |
| ACA | 99.29±9.36 | 9.42 | 98.31±6.83 | 1.94 | 105.96±2.06 | 1.94 | 103.5±4.22 | 4.08 | 101.45±12.19 | 12.01 | 106.13±3.75 | 3.53 |
| CA | 90.39±2.47 | 2.73 | 99.18±4.06 | 0.02 | 102.98±0.02 | 0.02 | 100.52±12.43 | 12.37 | 101.97±8.23 | 8.07 | 100.71±8.62 | 8.56 |
| TLCA-3S | 99.67±6.84 | 6.86 | 106.05±6.16 | 4.62 | 108.16±5 | 4.62 | 102.54±8.54 | 8.33 | 108.45±4.64 | 4.28 | 108.56±2.46 | 2.27 |
| LCA-3S | 101.18±10.4 | 10.27 | 106.33±9.55 | 5.46 | 108.43±5.92 | 5.46 | 102.47±5 | 4.88 | 108.14±3.43 | 3.17 | 110.35±1.18 | 1.07 |
| GLCA-3S | 101.08±5.08 | 5.02 | 110.47±3.25 | 3.29 | 103.63±3.41 | 3.29 | 98.97±9.43 | 9.53 | 99.74±10.7 | 10.73 | 103.89±1.19 | 1.15 |
| 7-ketoDCA | 99.3±10.38 | 10.45 | 98.42±4.02 | 6.26 | 98.83±6.19 | 6.26 | 104.16±7.13 | 6.84 | 101.35±10.89 | 10.74 | 102.8±9.37 | 9.11 |
| 3-oxoCA | 90.46±3.12 | 3.45 | 98.18±1.39 | 2.6 | 105.77±2.75 | 2.6 | 88.69±2.93 | 3.31 | 95.91±1.62 | 1.69 | 93.16±9.95 | 10.68 |
| DHCA | 99.1±13.42 | 13.54 | 103.45±15.38 | 3.35 | 109.04±3.65 | 3.35 | 91.81±3.47 | 3.78 | 93.48±9.76 | 10.44 | 105.88±7.42 | 7.01 |
| 6-ketoLCA | 99.29±9.58 | 9.65 | 105.04±8.17 | 3.87 | 106.84±4.13 | 3.87 | 102.43±2.04 | 1.99 | 97.69±11.12 | 11.38 | 98.79±8.51 | 8.62 |
| 7-ketoLCA | 98.85±9.56 | 9.67 | 99.51±3.14 | 2.95 | 109.16±3.22 | 2.95 | 97.4±10.7 | 10.99 | 98.28±11.91 | 12.11 | 108.49±1.66 | 1.53 |
| apoCA | 100.25±12.97 | 12.94 | 99.45±6.59 | 2.57 | 110.96±2.85 | 2.57 | 96.24±9.5 | 9.87 | 100.28±7.12 | 7.1 | 109.78±3.22 | 2.93 |
| LCA-d4 | 108.46±4.11 | 3.79 | 110.23±3.12 | 2.96 | 104.57±3.1 | 2.96 | 101.67±12.15 | 11.95 | 104.49±3.87 | 3.7 | 105.75±2.93 | 2.77 |
| CA-d4 | 108.68±5.06 | 4.65 | 101.04±2.83 | 0.87 | 106.27±0.92 | 0.87 | 95.94±8.76 | 9.13 | 104.23±1.46 | 1.4 | 103.82±1.32 | 1.27 |
| TCA-d4 | 99.41±3.77 | 3.79 | 106.77±4.03 | 5.21 | 103.11±5.37 | 5.21 | 91.78±6.43 | 7.01 | 107.3±5.61 | 5.23 | 101.39±7.83 | 7.72 |
| GCA-d4 | 104.99±2.98 | 2.84 | 98.45±11.19 | 4.18 | 104.31±4.36 | 4.18 | 103.93±7.61 | 7.32 | 110.29±4.15 | 3.77 | 108.17±5 | 4.62 |

**Table S9. Extraction recovery of 42 bile acid components from mouse serum and liver.**

| Bile acid | Serum Extraction Recovery | | | | | | Liver Extraction Recovery | | | | | |
| --- | --- | --- | --- | --- | --- | --- | --- | --- | --- | --- | --- | --- |
|  | LC (n = 6) | | MC (n = 6) | | HC (n = 6) | | LC (n = 6) | | MC (n = 6) | | HC (n = 6) | |
| isoalloLCA | 99.93±10.43 | 10.44 | 99.5±8.54 | 8.59 | 95.18±2.3 | 2.41 | 100.85±9.46 | 9.38 | 102.13±9.19 | 9 | 97.26±5.5 | 5.65 |
| isoLCA | 97.29±9.13 | 9.38 | 92.95±5.3 | 5.7 | 92.07±5.54 | 6.01 | 98.21±5.2 | 5.3 | 101.45±10.65 | 10.5 | 88.96±2.19 | 2.46 |
| LCA | 91.85±3.6 | 3.92 | 100.28±5.71 | 5.69 | 93.47±8.37 | 8.95 | 105.63±5.01 | 4.74 | 107.61±4.35 | 4.04 | 92.24±2.8 | 3.03 |
| UDCA | 102.37±9.63 | 9.41 | 105.97±3.03 | 2.86 | 93.77±5.44 | 5.8 | 93.72±8.72 | 9.31 | 101.9±1.53 | 1.5 | 101.21±5.99 | 5.92 |
| HDCA | 100.87±3.66 | 3.63 | 108.85±3.38 | 3.11 | 94.34±8.87 | 9.4 | 98.13±6.78 | 6.91 | 92.14±3.88 | 4.21 | 108.6±3.34 | 3.07 |
| CDCA | 99.78±5.18 | 5.19 | 105±4.54 | 4.33 | 90.22±6.41 | 7.1 | 104.54±3.98 | 3.81 | 88.85±1.9 | 2.14 | 97.2±4.44 | 4.57 |
| DCA | 104.14±3.76 | 3.61 | 100.18±6.95 | 6.94 | 91.26±5.49 | 6.01 | 98.51±9.81 | 9.96 | 97.14±8.38 | 8.62 | 99.33±5.08 | 5.12 |
| GLCA | 97.41±9.26 | 9.5 | 99.67±8.8 | 8.83 | 88.78±2.19 | 2.47 | 101.94±2.81 | 2.76 | 106.78±5 | 4.68 | 90.17±3.32 | 3.68 |
| GUDCA | 107±3.85 | 3.6 | 96.54±5.99 | 6.2 | 100.32±10.4 | 10.38 | 104.29±1.88 | 1.8 | 96.53±7.14 | 7.4 | 105.97±7.34 | 6.93 |
| GHDCA | 105.93±5.62 | 5.31 | 99.87±9.84 | 9.85 | 99.61±1.29 | 1.3 | 97.97±11.14 | 11.37 | 95.69±8.71 | 9.1 | 106.44±3.01 | 2.83 |
| GCDCA | 105.95±4.12 | 3.88 | 103.09±11.2 | 10.87 | 104.57±0.9 | 0.86 | 103.48±6.01 | 5.81 | 93.3±2.41 | 2.59 | 102.79±8.67 | 8.43 |
| GDCA | 89.75±8.24 | 9.01 | 102.26±7.75 | 7.58 | 109.86±2.36 | 2.15 | 105.22±1.72 | 1.63 | 94.11±7.46 | 7.93 | 104.25±4.23 | 4.06 |
| GDHCA | 94.53±9.24 | 9.77 | 96.71±11.41 | 11.79 | 87.94±2.73 | 3.11 | 95.2±2.75 | 2.89 | 90.96±3.89 | 4.27 | 95.2±0.73 | 0.76 |
| GHCA | 98.1±8.75 | 8.92 | 97.98±8.85 | 9.03 | 103.19±3.17 | 3.07 | 95.86±11.7 | 12.21 | 89.87±6.6 | 7.34 | 104.14±4.15 | 3.98 |
| GCA | 95.84±11.98 | 12.5 | 97.06±9.02 | 9.29 | 99.93±10.08 | 10.08 | 93.7±8.88 | 9.47 | 99.71±5.05 | 5.06 | 91.35±4.3 | 4.71 |
| TLCA | 102.2±5.61 | 5.49 | 108.62±3.62 | 3.33 | 95.93±6.35 | 6.62 | 103.34±6.9 | 6.68 | 96±9.29 | 9.67 | 98.51±6.92 | 7.02 |
| TUDCA | 102.03±10.8 | 10.59 | 98.37±3.38 | 3.44 | 98.64±6.19 | 6.27 | 95.98±10.7 | 11.15 | 90.37±1.83 | 2.02 | 105.31±5.19 | 4.93 |
| THDCA | 95.63±4.15 | 4.34 | 105.18±3.48 | 3.3 | 102.08±4.3 | 4.22 | 95.07±4.2 | 4.42 | 101.5±6.97 | 6.87 | 103.77±6.7 | 6.46 |
| TCDCA | 93.83±3.89 | 4.15 | 106.38±9.5 | 8.93 | 97.84±2.45 | 2.51 | 105.03±3.32 | 3.16 | 105.17±2.28 | 2.17 | 102.06±1.14 | 1.12 |
| TDCA | 101.2±10.23 | 10.11 | 98.57±5.2 | 5.27 | 97.32±4.44 | 4.56 | 98.84±8.02 | 8.11 | 97.06±9.2 | 9.48 | 103.26±7.76 | 7.52 |
| TDHCA | 93.69±10.02 | 10.69 | 98.77±9.14 | 9.25 | 98.98±9.11 | 9.2 | 96.92±6.34 | 6.55 | 96.67±11.26 | 11.65 | 96.44±7.09 | 7.35 |
| TαMCA | 107.54±3.06 | 2.84 | 104.32±12.07 | 11.57 | 102.51±7.2 | 7.02 | 101.44±9.29 | 9.16 | 103.25±1.57 | 1.52 | 102.38±3.12 | 3.05 |
| TβMCA | 105.06±5.51 | 5.24 | 105.51±5.68 | 5.38 | 101.22±3.43 | 3.38 | 98.77±3.06 | 3.1 | 105.89±2.65 | 2.5 | 102.22±3.02 | 2.95 |
| THCA | 98.45±8.11 | 8.24 | 104.32±6.52 | 6.25 | 101.01±1.68 | 1.66 | 101.37±9.87 | 9.74 | 95.53±5.14 | 5.39 | 102.47±5.2 | 5.07 |
| TCA | 101.77±7.7 | 7.56 | 105.22±6.28 | 5.97 | 99.58±4.97 | 4.99 | 108.18±3.8 | 3.52 | 105.04±3.05 | 2.91 | 106.07±4.56 | 4.3 |
| DHLCA | 94.13±4.96 | 5.27 | 93.76±8.26 | 8.81 | 94.49±5.75 | 6.08 | 98.83±7.9 | 7.99 | 95.31±8.8 | 9.23 | 88.34±1.3 | 1.47 |
| UCA | 93.46±5.34 | 5.72 | 105.09±9.63 | 9.17 | 100.3±5.23 | 5.22 | 98.39±5.28 | 5.37 | 104.4±3.34 | 3.2 | 107.84±4.98 | 4.62 |
| ωMCA | 101.62±5.06 | 4.98 | 105.49±3.65 | 3.46 | 98.45±4.99 | 5.07 | 100.82±8.93 | 8.86 | 103.46±2.9 | 2.81 | 103.57±3.43 | 3.31 |
| αMCA | 101.01±5.04 | 4.99 | 105.11±3.01 | 2.86 | 98.27±6.75 | 6.87 | 99.19±11.41 | 11.51 | 105.15±2.77 | 2.63 | 106.82±3.81 | 3.57 |
| βMCA | 106.01±2.84 | 2.68 | 105.77±4.77 | 4.51 | 95.74±6.41 | 6.7 | 104.83±2.67 | 2.54 | 98.62±5.96 | 6.04 | 98.96±3.78 | 3.82 |
| HCA | 98.49±10.71 | 10.88 | 103.32±3.48 | 3.37 | 98.55±6.71 | 6.81 | 97±10.22 | 10.53 | 106.56±2.72 | 2.55 | 102.51±3.95 | 3.85 |
| ACA | 99.76±5.61 | 5.62 | 101.68±7.88 | 7.75 | 91.7±2.8 | 3.06 | 96.6±9.6 | 9.94 | 102.02±8.51 | 8.34 | 91.66±4.71 | 5.14 |
| CA | 108.46±1.07 | 0.99 | 102.09±11.87 | 11.63 | 95.3±5.78 | 6.07 | 102.56±5.39 | 5.26 | 102.49±5.86 | 5.72 | 107.75±3.34 | 3.1 |
| TLCA-3S | 95.32±4.86 | 5.1 | 101.7±2 | 1.96 | 93.57±3.55 | 3.8 | 89.74±2.31 | 2.58 | 98.36±2.25 | 2.28 | 90.02±1.5 | 1.66 |
| LCA-3S | 89.91±3.35 | 3.73 | 95.21±6.47 | 6.79 | 90.37±4.66 | 5.15 | 88.71±5.65 | 6.37 | 92.09±5.29 | 5.75 | 90.68±1.7 | 1.87 |
| GLCA-3S | 91.86±9.55 | 10.4 | 104.21±9.64 | 9.25 | 95.41±9.13 | 9.56 | 94.38±6.85 | 7.25 | 98.87±4.17 | 4.22 | 88.7±2.34 | 2.64 |
| 7-ketoDCA | 98.53±7.26 | 7.37 | 102.21±4.26 | 4.17 | 98.75±11.97 | 12.12 | 103.34±6.43 | 6.23 | 106.05±2.52 | 2.38 | 100.92±4.84 | 4.8 |
| 3-oxoCA | 105.23±6.13 | 5.83 | 103.74±5.82 | 5.61 | 97.86±4.68 | 4.79 | 102.25±7.39 | 7.23 | 105.18±3.89 | 3.7 | 97.34±4.43 | 4.55 |
| DHCA | 97.92±11.69 | 11.93 | 98.41±11.74 | 11.93 | 88±2.93 | 3.33 | 96.57±8.64 | 8.95 | 92.31±6.25 | 6.77 | 91.73±3.8 | 4.15 |
| 6-ketoLCA | 101.39±4.29 | 4.24 | 104.42±2.56 | 2.45 | 92.36±4.54 | 4.92 | 104.4±10.61 | 10.17 | 103.88±1.95 | 1.87 | 95.33±1.72 | 1.8 |
| 7-ketoLCA | 97.16±6.21 | 6.39 | 108.8±5.21 | 4.78 | 91.55±7.74 | 8.46 | 98.4±8.08 | 8.21 | 104.29±3.6 | 3.45 | 92.91±4.32 | 4.65 |
| apoCA | 95.95±5.74 | 5.98 | 105.96±7.02 | 6.62 | 90.78±6.11 | 6.73 | 106.19±8.18 | 7.71 | 96.37±9.8 | 10.17 | 96.01±2.68 | 2.79 |
| LCA-d4 | 93.05±5.19 | 5.58 | 96.6±3.41 | 3.53 | 94.32±8.09 | 8.58 | 104.19±3.09 | 2.96 | 104±5.41 | 5.2 | 90.55±1.05 | 1.16 |
| CA-d4 | 100.25±7.68 | 7.67 | 104.47±6.45 | 6.18 | 96.15±2.99 | 3.11 | 104.86±2.72 | 2.59 | 91.96±5.71 | 6.21 | 100.03±7.41 | 7.41 |
| TCA-d4 | 100.2±12.76 | 12.73 | 102.26±2.77 | 2.7 | 101.18±7.34 | 7.25 | 104.88±4.38 | 4.17 | 95.39±1.54 | 1.61 | 101.85±9.61 | 9.43 |
| GCA-d4 | 96.29±10.87 | 11.29 | 92.23±9.7 | 10.51 | 94.58±4.86 | 5.14 | 94.44±9.84 | 10.42 | 95.67±9.05 | 9.46 | 90.69±6.59 | 7.26 |

**Table S10. Primers used for RT-PCR.**

| **Gene** | **Forward (5' to 3')** | **Reverse (5' to 3')** |
| --- | --- | --- |
| *Gapdh* | AGGTCGGTGTGAACGGATTTG | TGTAGACCATGTAGTTGAGGTCA |
| *Fxr* | TGGGTACCAGGGAGAGACTG | GTGAGCGCGTTGTAGTGGTA |
| *Creb* | AGCCGGGTACTACCATTC | GCTGCTTCCCTGTTCTTC |
| *Bdnf* | CCCATGAAAGAAGTAAACGTCC | CCTTATGGTTTTCTTCGTTGGG |
| *Oatp1a1* | CATCTTCTCACTCGCAGCCATCC | GGTGCCGGAATGCCAGCTAATAG |
| *Oatp1a4* | GGACTTCTGTTGGGATCTTCCTGTG | CAGCGTGTATCAGTGGGAGTTATGG |
| *Bsep* | GGACAATGATGTGCTTGTGG | CACACAAAGCCCCTACCAGT |
| Universal bacteria | CGGTGAATACGTTCCCGG | TACGGCTACCTTGTTACGACTT |
| *P. distasonis* | GAGTATGTTTGAGGCAGGCG | CGATACACTGTAAGCGGCAC |

**Table S11. Top 10 bile acids changed in bile acid manipulation and FMT rescue experiments.**

| **Bile acid manipulation experiment** | | | | | | | **FMT rescue experiment** | | | | | | | | | | | |
| --- | --- | --- | --- | --- | --- | --- | --- | --- | --- | --- | --- | --- | --- | --- | --- | --- | --- | --- |
| **NC vs CSS** | | | | **CSS+CHOL vs CSS+CHOL+CA** | | | **NC vs CSS** | | | | **CSS+CHOL vs CSS+CHOL+FMT** | | | | **FMT vs FFMT** | | | |
| **BA** | | **log2**  **(FC)** | **-log10 Pvalue** | **BA** | **log2**  **(FC)** | **-log10 Pvalue** | **BA** | **log2**  **(FC)** | **-log10 Pvalue** | **BA** | | **log2**  **(FC)** | **-log10 Pvalue** | **BA** | | **log2**  **(FC)** | **-log10 Pvalue** |  |
| 7-ketoDCA | 3.37 | | 10.3 | 7-ketoDCA | 2.5 | 2.09 | HCA | 2.47 | 3.67 | TβMCA | | 3.22 | 1.33 | UCA | | 3.53 | 0.63 |  |
| HCA | 2.86 | | 5.9 | DCA | 1.67 | 2 | 7-ketoDCA | 2.21 | 2.7 | THDCA | | 2.54 | 0.77 | 7-ketoDCA | | 2.94 | 1.55 |  |
| 6-ketoLCA | 2.57 | | 2.48 | TDCA | 1.5 | 2 | UCA | 1.37 | 1.3 | ωMCA | | 2.4 | 1.46 | 3-oxoCA | | 2.37 | 0.94 |  |
| THDCA | 1.94 | | 4.95 | 3-oxoCA | 1.2 | 1.68 | ωMCA | 1 | 1.08 | βMCA | | 2.12 | 2.48 | DHLCA | | 1.44 | 0.74 |  |
| 3-oxoCA | 1.89 | | 4.88 | HCA | -1.18 | 0.67 | CA | 0.99 | 1.2 | TUDCA | | 1.88 | 0.86 | TDCA | | 1.13 | 1.77 |  |
| CDCA | 1.75 | | 7.59 | βMCA | -1.12 | 0.84 | βMCA | 0.99 | 1.13 | UDCA | | 1.71 | 1.98 | LCA | | 1.1 | 0.93 |  |
| βMCA | 1.72 | | 5.73 | 6-ketoLCA | -1.06 | 0.64 | apoCA | 0.85 | 0.56 | HDCA | | 1.6 | 1.28 | HCA | | 1.09 | 2.19 |  |
| CA | 1.71 | | 5.03 | THDCA | -1.06 | 1.14 | αMCA | 0.57 | 0.43 | 7-ketoDCA | | 1.52 | 1.64 | CA | | 1.05 | 0.83 |  |
| THCA | 1.6 | | 1.55 | TβMCA | -0.99 | 0.69 | DHLCA | 0.55 | 0.85 | αMCA | | 1.27 | 2.07 | 7-ketoLCA | | 0.8 | 0.88 |  |
| HDCA | 1.57 | | 3.19 | αMCA | -0.82 | 0.65 | 6-ketoLCA | 0.51 | 0.32 | HCA | | 1.08 | 1.75 | DCA | | 0.78 | 1.11 |  |

**Table S12. Fold change of liver BAs for the CSS versus NC group in bile acid manipulation experiment.**

| **BA** | **log2(FC)** | **-log10(Pvalue)** |
| --- | --- | --- |
| 7-ketoDCA | 1.536865891 | 1.147177311 |
| THDCA | 0.910949256 | 1.063226535 |
| THCA | 0.792835131 | 0.794280275 |
| 6-KETOLCA | 0.549441202 | 0.478412013 |
| HDCA | 0.264213753 | 0.17967169 |
| HCA | 0.209126673 | 0.131681435 |
| GCA | 0.190967175 | 0.124171615 |
| TCA | 0.155806668 | 0.123870236 |
| TDCA | 0.11441485 | 0.06922908 |
| TbMCA | 0.023498216 | 0.018720749 |
